# Counteracting Protein Kinase Activity in the Heart: The Multiple Roles of Protein Phosphatases

**DOI:** 10.3389/fphar.2015.00270

**Published:** 2015-11-13

**Authors:** Silvio Weber, Stefanie Meyer-Roxlau, Michael Wagner, Dobromir Dobrev, Ali El-Armouche

**Affiliations:** ^1^Department of Pharmacology and Toxicology, Dresden University of Technology, Dresden, Germany; ^2^Institute of Pharmacology, Faculty of Medicine, West German Heart and Vascular Center, Essen, Germany

**Keywords:** PP1, PP2A, calcineurin, PIP, protein phosphatase inhibitor-1 (I-1)

## Abstract

Decades of cardiovascular research have shown that variable and flexible levels of protein phosphorylation are necessary to maintain cardiac function. A delicate balance between phosphorylated and dephosphorylated states of proteins is guaranteed by a complex interplay of protein kinases (PKs) and phosphatases. Serine/threonine phosphatases, in particular members of the protein phosphatase (PP) family govern dephosphorylation of the majority of these cardiac proteins. Recent findings have however shown that PPs do not only dephosphorylate previously phosphorylated proteins as a passive control mechanism but are capable to actively control PK activity via different direct and indirect signaling pathways. These control mechanisms can take place on (epi-)genetic, (post-)transcriptional, and (post-)translational levels. In addition PPs themselves are targets of a plethora of proteinaceous interaction partner regulating their endogenous activity, thus adding another level of complexity and feedback control toward this system. Finally, novel approaches are underway to achieve spatiotemporal pharmacologic control of PPs which in turn can be used to fine-tune misleaded PK activity in heart disease. Taken together, this review comprehensively summarizes the major aspects of PP-mediated PK regulation and discusses the subsequent consequences of deregulated PP activity for cardiovascular diseases in depth.

## Introduction

Myriads of studies starting from the late 1930s have unequivocally shown that protein kinases (PKs) are essential for cellular homeostasis not only in the heart but in virtually any tissue of the body (Cori et al., 1939; Rapundalo, 1998; Cohen, 2002a; Harvey, 2004; Johnson, 2009; Sato et al., 2015). We have now gathered a deep understand how PKs work in the context of cardiovascular diseases (CVDs) and consequently it is anticipated that PKs may serve as the pharmacological drug target of the twenty-first century (Cohen, 2002a,b; Force et al., 2004; Belmonte and Blaxall, 2011; Roskoski, 2015). Although discovered at an equally early time point in scientific history, protein phosphatases (PPs) have received much less attention in terms of functional studies and for consideration as potential drug targets. This is somewhat astonishing as (phospho-)proteomic studies suggested that nearly one third of all protein phosphorylation events is reversible (Sefton, 2001; Olsen et al., 2006) and it seems likely that targeting phosphatases in CVDs can be similarly promising. One explanation for this observation can be certainly attributed to the difference in substrate selection between PKs and PPs (Brinkworth et al., 2003; Zhu et al., 2005; Ubersax and Ferrell, 2007; Roy and Cyert, 2009; Slupe et al., 2011; Li et al., 2013; Peti et al., 2013; Palmeri et al., 2014). While more than 400 available PKs often recognize decent consensus sequences in their target proteins, a far smaller number of phosphatases does not rely on specific enzyme-substrate recognition but mainly interact via so called phosphatase interacting proteins (PIPs) with their respective substrate, thus decreasing druggability enormously in comparison to PKs (Cohen, 2002c; Chatterjee and Kohn, 2013; De Munter et al., 2013; Li et al., 2013). Nevertheless recent developments have revealed exciting opportunities for the application of phosphatase-regulating drugs in CVDs and will therefore be discussed at the end of this review.

**FIGURE 1:**
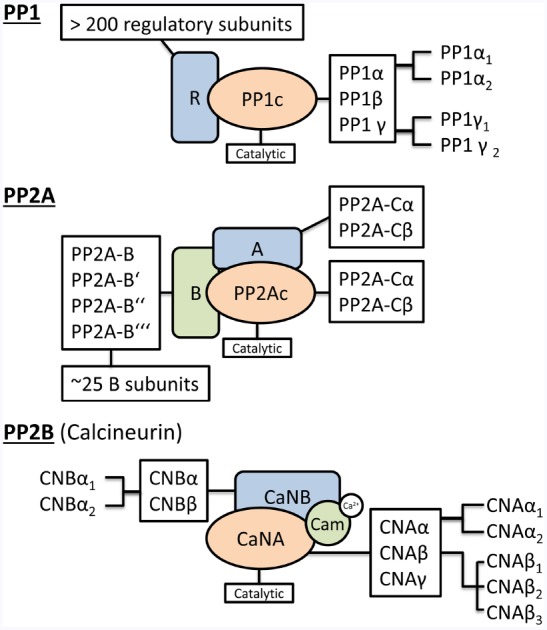
**Holoenzyme composition of protein phosphatases.** The schematic drawing of the holoenzyme composition of PP1, PP2A and PP2B (Calcineurin) indicates the combinatorial complexity of the different catalytic and regulatory protein phosphatase subunits. A detailed description of the underlying nomenclature and the nature of the regulatory subunits can be found in Heijman et al. (2013).

Protein phosphatases can be divided into three different subgroups, namely serine-threonine, tyrosine, and dual-specific phosphatases depending on their endogenous phosphorylation substrate (Maillet et al., 2008; Auger-Messier et al., 2013; Heijman et al., 2013; Senis, 2013). Thereby more than 98% of dephosphorylation events are carried out by serine-/threonine phosphatases and within this group PP 1, 2A, and 2B (PP1, PP2A and PP2B, also called calcineurin) amount to 90% of all dephosphorylation activity in the heart (Cohen, 1989; MacDougall et al., 1991). As a consequence this review will focus on the latter PPs and their role as cellular opponents of the aforementioned PKs in the healthy and diseased heart. Nevertheless we would like to refer the reader to some recent excellent reviews and research publication showing the physiological and pathophysiological importance of phosphatases which are not discussed in this review (Pulido and Hooft van Huijsduijnen, 2008; Patterson et al., 2009; Senis, 2013; Lauriol et al., 2015). Before we will deeply dive into the functional role of PP1, PP2A, and PP2B and their potential as drug target in CVDs, we will shortly recapitulate the structure of these enzymes which are virtually all working as holo-enzymes as this is crucial for the understanding of the following chapters. Figure 1 shows a schematic drawing of the mouse representative PP1, PP2A, and PP2B holoenzymes known so far.

In mammals, the catalytic PP1 subunit is encoded by three separate genes (PPP1CA, PPP1CB, and PPP1CC) which seem to have distinct subcellular functions as suggested by initial studies using isoform specific knock-down and knock-out studies (Aoyama et al., 2011; Liu et al., 2015). Nevertheless there is neither detailed information available about spatiotemporal control of PP1 isoforms in the healthy or diseased heart nor insights into the molecular mechanism at this time (Luss et al., 2000). Further diversification of PP1 isoforms is achieved by alternative splicing of PPP1CA (PP1α_1–3_) and PPP1CC (PP1γ_1/2_). PP1γ_2_ seems to be the only tissue-specific expressed PP1 (brain and testis) while all other isoforms are expressed ubiquitously (Cohen, 2002c; Ceulemans and Bollen, 2004; Korrodi-Gregorio et al., 2014). Multiple studies have suggested that there is actually no freely available PP1 within the cardiac cell but rather a competition of >150 regulatory subunits to form a holo-complex with the PP1 catalytic subunits. These regulatory subunits determine subcellular localization and substrate specificity of the different PP1 isoform catalytic subunits. A series of excellent reviews gives a comprehensive overview about this aspect of PP1 (Ceulemans and Bollen, 2004).

The composition of the PP2A holo-enzyme is even more complex. It can be either a heterodimer consisting of the catalytic (PP2A-Cα or Cβ) and the structural scaffold (PP2A-Aα or Aβ) or a trimer consisting of the catalytic, the structural scaffold and another regulatory subunit (PPP2RX; Herzig and Neumann, 2000; Heijman et al., 2013). The importance of PP2a regulation becomes obvious since altered expression and activities are closely associated with heart diseases (Lei et al., 2015). Intriguingly PP1 and PP2A seem to share a couple of substrates together (Heijman et al., 2013). If this is due to compensatory mechanisms after genetic knockdown or knockout of single PP1/PP2A genes or caused by lack of specificity of the available pharmacological inhibitors has still to be determined. For further reading about the molecular mechanisms underlying PP2A regulation in the heart we would again like to recommend another set of enlightening reviews (Janssens and Goris, 2001; DeGrande et al., 2013; Lei et al., 2015).

Finally we would like to highlight PP2B (or calcineurin) as an essential cardiac PP which links Ca^2+^ and phosphorylation-dependent signaling pathways. Initially mainly studies identified calcineurin as a regulator of Ca^2+^ mediated gene transcription during cardiac remodeling, but recently calcineurin was also shown to directly act as an essential enzyme for reversible cardiac protein phosphorylation, e.g., at the cardiac L-Type Ca^2+^-channel (Wang et al., 2014). Heart-restricted calcineurin overexpression in mice lead to cardiac remodeling, arrhythmic events and premature, sudden cardiac death (Molkentin, 2000). Again, PP2B is mainly active as a holo-enzyme consisting of one of the three different catalytic isoforms CNAα, β or γ, the Ca^2+^-binding subunit (CNB_α/β_) and sometimes another regulatory protein, e.g., AKAP or Cain (Lim and Molkentin, 1999; Wolska, 2009; Heineke and Ritter, 2012; Wang et al., 2014).

After clarifying the functional impact and physiological importance of PPs in the heart, we will now move on to discuss which cardiac proteins are targets of reversible protein phosphorylation by PKs and PPs and how this may relate to CVD pathology.

## Protein Phosphatases Counteract Protein Kinase Mediated Protein Phosphorylation in the Heart

**FIGURE 2:**
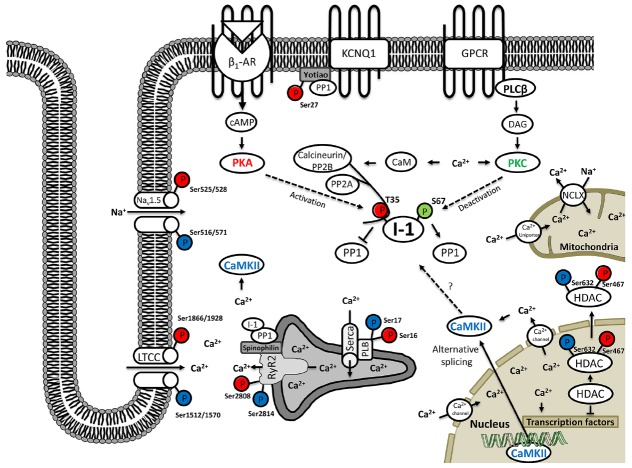
**Protein phosphatase inhibitor-1 (I-1) fulfills a nodal role during reversible protein phosphorylation in cardiomyocytes.** On the primary level I-1 is mainly regulated by the complex interplay of protein kinases: PKA (red color), PKC (green color) and indirectly also CaMKII (blue color) vs. protein phosphatases 2A and 2B. While PKA (and CaMKII) have activating function, PKC, PP2A, and PP2B are limiting I-1 activity. On the secondary level, CaMKII and PP2B and thus subsequently also I-1, are regulated by intracellular calcium levels, which are mediated by a complex flux balance from internal Ca^2+^ stores of the sarcoplasmic reticulum, the mitochondria and the nucleus as well as Ca^2+^ influx from extracellular space. In turn I-1 is mainly acting on protein phosphatase 1 which dephosphorylates a multitude of functional proteins of the cardiomyocyte (see Table 1) and can act on different cellular functions including contraction, ion flux/currents, transcription and/or splicing, accordingly.

One of the hallmarks of CVDs is altered phosphorylation of cardiac proteins (Rapundalo, 1998; Luo and Anderson, 2013). Thereby most of the knowledge stems from studies of isolated cardiac myocytes, while studies about the substrates of reversible phosphorylation in cardiac fibroblasts, endothelial and smooth muscle cells are rather limited. Table 1 gives a comprehensive and compartment-sorted overview about PP1, PP2A, and PP2B substrates in cardiac cells. In this chapter we highlight some important examples of phosphatase-dependent substrate regulation in cardiac cells and visualize them additionally in Figure 2.

**TABLE 1:** **Targets for reversible cardiac protein phosphorylation**.

**Protein**	**Reported kinase activity**	**Phosphorylation site**	**Reported phosphatase activity**	**Reference**
Aurora B	n.d.	n.d.	PP1	Murnion et al. (2001)
Ca_V_1.2 α	CaMKII	Ser1512, Ser1570	PP1 PP2A PP2B	Davare et al. (2000), Kamp and Hell (2000), Chen et al. (2002), Yang et al. (2005, 2007), Hall et al. (2006), Hulme et al. (2006), Lee et al. (2006), Sun et al. (2006), Lemke et al. (2008), Tandan et al. (2009), Blaich et al. (2010), Xu et al. (2010), Brandmayr et al. (2012), Shi et al. (2012), Minobe et al. (2014)
	PKA	Ser1574, Ser1866, Ser1928	PP1 PP2A	
PKC	Ser1928	PP1 (major) PP2A (minor)	
PKG	Ser1928	
Akt/PKB	n.d.	n.d.
Ca_V_1.2 β	PKC	Ser496	PP1 PP2A	Gerhardstein et al. (1999), Grueter et al. (2006), Yang et al. (2007), Abiria and Colbran (2010)
	CaMKII	Thr498	
PKA	Ser458, Ser478, Ser479	
PKG	Ser496	
cMyBP-C	PKAPKC	Ser282 Ser273 (HF) Ser302 (HF)	PP1 PP2A	Mohamed et al. (1998), Florea et al. (2012), Heijman et al. (2013)
Cold shock domain protein ACSDA	Bcr-Abl kinasePI3K/AktRibosomal S6 kinase (RSK)	Ser134	PP1	Sears et al. (2010), Chiang et al. (2015)
Connexin 43	PKC	Ser368	PP1 PP2A	Ai and Pogwizd (2005), Ai et al. (2011)
	PKA	Ser262	
CREB	PKA PKC Casein kinase II CaMKIIP90 (RSK)	Ser133	PP1 PP2A	Montminy et al. (1990), Miller et al. (1998), Li et al. (2006)
eIF2α	n.d.	n.d.	PP1	Ceulemans and Bollen (2004)
FAK	n.d.	n.d.	PP1α *in vitro*	Yamakita et al. (1999)
Glycogen synthase kinase	PKA	Ser67 Ser48	PP1 PP2A	Woodgett and Cohen (1984), Walker et al. (2000)
HDAC4	CKIP PKA CaMKII	Ser246 Ser467 Ser632	PP2A	Zhang et al. (2002), Backs et al. (2006)
Histone3	CaMKII	Thr3 Ser28 Ser10	PP1	Ceulemans and Bollen (2004), Awad et al. (2013)
I-1	PKA	Thr35	PP2A PP2B	El-Armouche et al. (2006a), Wittkopper et al. (2010a)
	PKCα	Ser67	
Na^+^/K^+^ ATPase	PKC	Ser18	PP2A	Palmer et al. (1991), Feschenko and Sweadner (1997)
Na_V_1.5	CaMKII	Ser571 Thr594 Ser516	n.d.	Murray et al. (1997), Zhou et al. (2000), Baba et al. (2004), Wagner et al. (2006), Hund et al. (2010), Ashpole et al. (2012), Toischer et al. (2013)
	PKA	Ser525 Ser528	
	PKC	n.d.	
NCX1	PKA (*in vitro*), PKC	Thr731 (*in vitro*)	PP1 PP2A PP2B	Palmer et al. (1991), Schulze et al. (2003), Wei et al. (2003, 2007), Shigekawa et al. (2007), Zhang and Hancox (2009), Wanichawan et al. 2011
Neurabin	P70S6 kinase	n.d.	PP1	McAvoy et al. (1999), Sakisaka et al. (1999), Oliver et al. (2002)
	PKA	Ser461	
NFATs	PKA GSK3β DYRK1/2 CK1 MEKK1 JNK P38MAPK	multiple	PP2B	Beals et al. (1997), Chow et al. (1997, 2000), Zhu et al. (1998), Chow and Davis (2000), Gomez del Arco et al. (2000), Sheridan et al. (2002), Yang et al. (2002), Okamura et al. (2004), Gwack et al. 2006
PDE4D3	PKA	Ser13 Ser54	PP1 PP2A	Egloff et al. (1997), Carlisle Michel et al. (2004), Dodge-Kafka et al. (2006), De Arcangelis et al. 2008
	ERK5	Ser579	
PDE5A	n.d.	n.d.	PP1	Chiang et al. (2015)
Phospholemman	PKA	Ser68	PP1	Palmer et al. (1991), El-Armouche et al. (2011)
	PKC	Ser63 Ser68	
PLB	PKA	Ser16	PP1 PP2A	MacDougall et al. (1991), Luo et al. (1994), Jackson and Colyer (1996), Chu and Kranias (2002)
	CaMKII	Ser17	
PMCA	PKA PKC	n.d.	PP1 PP2A	Zylinska et al. (1998), Zylinska and Soszynski (2000)
P70S6 Kinase	n.d.	n.d.	PP1 PP2A	Bettoun et al. (2002)
Retinoblastoma protein (Rb)	CDK	Thr320	PP1	Liu et al. (1999), Ceulemans and Bollen (2004)
RyR2	PKA	Ser2808 Ser2030	PP1 PP2A PP2B	Marx et al. (2000), Pare et al. (2005), Xiao et al. (2005, 2006), Meng et al. (2007), Liu et al. (2011, 2014), Zhang et al. (2012)
	CaMKII	Ser2814	
SERCA2a	CaMKII	Ser38 (Ser167, Ser531 → *in vitro*)	n.d.	Brandl et al. (1986), Narayanan and Xu (1997)
SF2/ASF	PKA	n.d.	PP1	Gu et al. (2011), Huang et al. (2014)
Tn-inhibitor (TnI)	PKA	Ser23 Ser24	PP1 PP2A	Cole and Perry (1975)
T-type calcium channel (Cav3.2)	PKA	Ser1107 Thr2214 Ser1144	n.d.	Chemin et al. (2007), Hu et al. (2009)
Vitamin D receptor	P70S6PI-3 kinaseB/Akt	n.d.	PP1 PP2A	Bettoun et al. (2002)
Yotiao/KCNQ1	PKA	Ser27	PP1	Westphal et al. (1999), Marx et al. (2002), Terrenoire et al. (2009)

This table lists currently known substrates, which PK-mediated phosphorylation is counteracted by PP1, PP2A, or/and PP2B.

### The L-Type Calcium Channel, Na^+^/K^+^-ATPase and Other Ion Channels—Reversible Protein Phosphorylation at the Plasma Membrane

Reversible phosphorylation has been shown for a huge subset of different ion channels and their respective subunits. While the kinases required for phosphorylation have often been identified, the exact nature and contribution of phosphatases remained enigmatic due to the aforementioned reasons. The best studied examples of reversible protein phosphorylation at the plasma membrane are therefore the L-Type calcium channel (Ca_V_1.2) and the Na^+^/K^+^-ATPase dependent substrates in cardiac cells. Phosphorylation at the α-Subunit of Ca_V_1.2 takes place on multiple residues including Ser1512 and Ser1570 (CaMKII) as well as Ser1866 and Ser1928 (PKA; Chen et al., 2002; Yang et al., 2005; Lee et al., 2006; Lemke et al., 2008; Blaich et al., 2010; Minobe et al., 2014). There is still controversy which of these phosphorylation sites is the most important one for regulation of the Ca_V_1.2 function, but most studies hint at an essential role of Ser1928 (Yang et al., 2005; Hulme et al., 2006; Lemke et al., 2008; Xu et al., 2010). It seems as if PP1, PP2A, and even PP2B can bind to this region and regulate dephosphorylation levels in concert (Davare et al., 2000; Hall et al., 2006; Tandan et al., 2009; Shi et al., 2012). It was reported that PP1 and PP2A act directly on the phosphorylation level of Ca_V_1.2 and PP2B may have rather indirect effect by blocking PP1 and PP2A binding sites on the one hand and controlling Ca_V_1.2 expression level via the CREB signaling pathway on the other hand (Tandan et al., 2009; Wang et al., 2014). Spatiotemporal control of PP2A activity at the Ca_V_1.2 alpha-subunit is known to be mediated via the B56 (all isoforms) or PR59 subunits, while the mechanism of regulation of PP1 is still unknown (Hall et al., 2006; Xu et al., 2010). In general, PP2A-mediated dephosphorylation leads to downregulated Ca_V_1.2 activity (Heijman et al., 2013). Finally pharmacological inhibition studies suggested that aforementioned PPs may play a role for dephosphorylation of basal as well as adrenoceptor induced Ca_V_1.2 phosphorylation, which renders them different from PKA and CaMKII, that were shown to be either more important for basal (CaMKII) or adrenoceptor induced (PKA) Ca_V_1.2 phosphorylation (Brandmayr et al., 2012). As tight control of Ca_V_1.2 activity during EC-coupling is essential and deregulated Ca_V_1.2 function has been shown in a multitude of different CVDs, e.g., hypertension, cardiac arrhythmia and heart failure, specific targeting of PP-Ca_V_1.2 interactions might be a versatile drug targeting in the future (Splawski et al., 2004; Tang et al., 2008; Domes et al., 2011; Hong et al., 2012; Tajada et al., 2013).

Another important target for reversible dephosphorylation at the plasma membrane is the Na^+^/K^+^-ATPase, which can be either indirectly regulated via PP1-mediated dephosphorylation of the Na^+^K^+^-ATPase subunit phospholemman at position Ser68 or by direct recruitment of PP2A via its subunit PP2A-B56 and subsequent dephosphorylation at position Ser18 (Neumann et al., 1999; El-Armouche et al., 2006a; Bhasin et al., 2007). Interestingly binding of PP2A to Na^+^K^+^-ATPase diminishes binding of arrestin, thus increasing membrane localization of the pump and thereby actively modulating function of the Na^+^K^+^-ATPase (Kimura et al., 2011). The above mentioned studies also provided an important breakthrough in better understanding the role of reversible Na^+^K^+^-ATPase phosphorylation for CVD development. The described mechanism is markedly perturbed in failing hearts favoring phospholemman dephosphorylation and Na^+^K^+^-ATPase deactivation and thus may contribute to maladaptive hypertrophy and arrhythmogenesis via chronically increased intracellular Na^+^ and Ca^2+^ concentrations (Neumann et al., 1999; Schwinger et al., 2003; Cheung et al., 2010; El-Armouche et al., 2011).

Finally there are some reports indicating reversible phosphorylation of Sodium and Potassium Channels, e.g., Na_V_1.5 and KCNQ1, that are essential for membrane excitation (Murray et al., 1997; Terrenoire et al., 2009; Marionneau et al., 2012; Toischer et al., 2013). In case of Na_V_1.5 many different phosphorylation sites have been described, with a priority of PKA-mediated phosphorylation in peak I_Na_ and CamKII-mediated phosphorylation during late I_Na_ (Baba et al., 2004; Bers and Herren, 2012; Marionneau et al., 2012). To our knowledge there is just one report about a protein tyrosine phosphatase (PTP1) working as a counteracting phosphatase after PKA or CamKII-mediated Na_V_1.5 phosphorylation, so additional studies to identify putative PP-mediated Na_V_1.5 dephosphorylation might be helpful (Jespersen et al., 2006). Even less is known with respect to reversible KCNQ1 phosphorylation. Initial studies show that PKA can mediated phosphorylation of KCNQ1 at position Ser27 at its C-terminal tail, which may by counteracted by PP1 and regulated by the accessory protein Yotiao (Marx et al., 2002; Nicolas et al., 2008). However, more studies are needed to understand the functional implication of reversible phosphorylation in this macromolecular protein complex at the plasma membrane are needed.

### Phospholamban and the Ryanodine Receptor—Reversible Protein Phosphorylation at the Sarcoplasmic Reticulum

Disturbed Ca^2+^-handling is coming along with most CVDs and numerous studies using genetically modified or pharmacologically treated animals have revealed the importance of tight spatiotemporal control of the two sarcoplasmic reticulum (SR) residing proteins ryanodine receptor type-2 (RyR2) and phospholamban (PLB) in this context. Consequently, tremendous efforts have been undertaken to identify the exact contribution of kinases and phosphatases toward deregulated phosphorylation of the latter proteins. While the large intracellular C-terminus of RyR2 contains multiple putative phosphorylation sites, only three of them have been extensively studied until today. Although still under debate, it seems as if Ser2808 and Ser2030 are the major PKA and Ser2814 is the major CaMKII phosphorylated site (Marx et al., 2000; Marks et al., 2002; El-Armouche and Eschenhagen, 2009). Dephosphorylation can be carried out by either PP1, which is targeted by spinophilin and/or PP2A which is targeted by its subunit PR130 to the multiprotein RyR2 holo-complex (Marx et al., 2001; Reiken et al., 2003; Chiang et al., 2014). There are no reports about calcineurin mediated RyR2 dephosphorylation but it has been suggested that deregulated RyR2 activity can modulate calcineurin expression in turn, thus serving as a potential hub for feedback inhibition of reversible cardiac protein phosphorylation (Zou et al., 2011).

In contrast PP1 is the major dephosphorylating enzyme of PLB, which critically modulates the activity of the SR Ca^2+^-ATPase (SERCA2a). A small contribution has been also shown for PP2A, but this relates to less than 30% of the dephosphorylation events at positions Ser16 (PKA-controlled) and Thr17 (CaMKII-controlled; MacDougall et al., 1991; Jackson and Colyer, 1996; Chu and Kranias, 2002; Aye et al., 2012). Increased targeting of PP1 to PLB is achieved by R_GL_ or HSP20, which leads to reduced SERCA2a Ca^2+^ uptake and thus attenuated β-AR stimulation-induced lusitropy (Berrebi-Bertrand et al., 1998).

### Troponin-I and cMyBP-C—Reversible Protein Phosphorylation at the Myofilaments

The most important consequences of the elevated systolic Ca^2+^-level is the activation of the contractile apparatus, which is again dependent on the amount of phosphorylation of the associated contractile proteins, e.g., the cardiac myosin-binding protein-C (cMyBP-C) or the Inhibitor of the Troponin complex, Troponin-I (Barefield and Sadayappan, 2010). For the latter, PP1 seems to preferentially dephosphorylate Ser23 and Ser24 on TnI, while the exact substrate of PP2A is yet undetermined (Jideama et al., 2006). Nevertheless it could be detected that targeting of PP2A is B56α-dependent and β-AR stimulation seems to be able to dissociate this localized interaction (Yin et al., 2010).

Phosphoproteomic studies revealed a number of putative phosphorylation sites also for cMyBP-C (Solaro and Kobayashi, 2011). Interestingly reduced phosphorylation at position Ser282 coincides with increased PP1 and PP2A activity in patients with atrial fibrillation (El-Armouche et al., 2006b). Similar results were received in end-stage heart failure, however here phosphorylation was also diminished at positions Ser273 and Ser302 (El-Armouche et al., 2007b; Sumandea and Steinberg, 2011).

### I-1 and PDE4D3—Reversible Protein Phosphorylation in the Cytoplasm

An excellent showcase for the description of a typical substrate for reversible phosphorylation of a cardiac protein represents the PP1-specific inhibitor of protein phosphatase-I (I-1). I-1 fulfills the function as a central signaling hub in feedback control of phosphatase activity and is thus topic of a number of reviews itself (Nicolaou et al., 2009; Wittkopper et al., 2010a, 2011). I-1 activity depends on PKA-dependent Thr35 phosphorylation, which seems to be mediated via AKAP18 (Endo et al., 1996; Singh et al., 2011). During β-AR stimulation, PKA-controlled I-1 activation and subsequent PP1 inhibition form a positive feed-back loop amplifying the phosphorylation of substrates like PLB, RyR2, and phospholemman (Carr et al., 2002; El-Armouche et al., 2003). Dephosphorylation of I-1 at position Thr35 is carried out by PP2A and PP2B, thus creating a crosstalk between the different phosphatases as well as between PKA and Ca^2+^-related second-messenger systems (via PP2B mediated dephosphorylation of I-1; El-Armouche et al., 2006a). Moreover, I-1 is also phosphorylated by PKCα on Ser67, this time resulting in reduced I-1 activity and decreased PLB phosphorylation. Conversely, the Ser67Ala substitution, preventing inactivation, increases PLB phosphorylation (Huang and Paudel, 2000; Braz et al., 2004). Figure 1 gives a schematic overview about the nodal role of I-1 during reversible cardiac protein phosphorylation.

Much less is known about reversible phosphorylation of phosphodiesterases, another important players of the adrenergic response. PP2A could be shown as a negative regulator of PDE4D3 activity in a complex with mAKAP, RyR2 and PKA (Carlisle Michel et al., 2004; Dodge-Kafka et al., 2006; De Arcangelis et al., 2008). In this case PP2A activity is itself controlled by PP2B (Dodge-Kafka et al., 2010). Further studies will be needed to elucidate the contribution of reversible phosphorylation toward function of cardiac PDEs, as malfunction of this enzymes can be beneficial or detrimental in multiple CVDs.

### Histone 3 and HDAC4—Reversible Protein Phosphorylation in the Nucleus

Several studies have implicated localization and activity of phosphatases in different compartment of the nucleus including chromatin bound fractions (Bollen and Beullens, 2002; Moorhead et al., 2007). There is extensive knowledge about the counteracting role of PPs against PKs during different phases of the cell cycle in non-cardiac cells (Ceulemans and Bollen, 2004; Novak et al., 2010; Jeong and Yang, 2013). As adult cardiac myocytes are mainly non-dividing cells, phosphatase targets may be different and up to this point there are only sparse reports about nuclear phosphatase targets in this regard (von Holtey et al., 1996; Heineke and Ritter, 2012). An exception hereof is histone deacetylase 4 (HDAC4), which phosphorylation status was well studied. While CKIP and CaMKII seem to be the main kinases phosphorylating Ser246, and Ser632, PP2A was identified as a major phosphatase to control HDAC4 phosphorylation status and subsequent subcellular localization, thus modulating HDAC4 activity (Paroni et al., 2008; Ling et al., 2012; Liu and Schneider, 2013; Kreusser and Backs, 2014). Knockdown experiments of PP1 did not reveal a significant contribution toward HDAC4 dephosphorylation. This might however be different for another important phosphatase substrate of the nucleus, which is histone 3 (H3). Phosphorylation of H3 occurs at multiple positions, including Ser10 and Ser28 (Awad et al., 2013; Korrodi-Gregorio et al., 2014). PP1 was reported to significantly contribute toward dephosphorylation of at least Ser10 in non-cardiac cells rendering it a potential target also in cardiac myocytes (Awad et al., 2013). In summary PPs may act as important and rapid modifiers of the epigenetic code and predispose DNA and protein sequences for subsequent methylation—a concept that is also known as the methyl-phospho-switch hypothesis (Chen et al., 2008; Sawicka and Seiser, 2014).

### Nuclear Factors of Activated T-Cells (NFATs)—Regulation of Transcription by Ca^2+^-dependent Dephosphorylation

Nuclear factors of activated T-cells (NFATs) are a family of transcription factors in which the nuclear localization signals (NLS) are masked by multiple phosphorylations in their serine-rich domains. Upon activation of PP2B by Ca^2+^/calmodulin, they are dephosphorylated, leading to their nuclear translocation, initiation of transcription and induction of cardiac hypertrophy as well as electrical remodeling (Heineke and Molkentin, 2006; Heineke and Ritter, 2012). A potential role in cardiac hypertrophy has been shown for NFATc2, NFATc3, and NFATc4. A number of PKs have been inflicted in cardiac NFAT phosphorylation, among them PKA, GSK3β, DYRK1/2, CK1, MEKK1, JNK and P38MAPK, and some isoform specificity is present. Importantly, PKA and DYRK1/2 can act as priming kinases, enabling other kinases to access the NFAT phosphorylation sites (Pan et al., 2013). The importance of PP2B/NFAT signaling in cardiac hypertrophy was first recognized in 1998, when Molkentin et al. (1998) demonstrated that overexpression of activated PP2B of NFATc4 lead to cardiac hypertrophy. Later it became clear that NFATc3 as well as c4, but not c2 are necessary for cardiac hypertrophy in multiple models (Wilkins et al., 2004; Bourajjaj et al., 2008). Moreover, PP2B/NFAT signaling plays an important role in shaping physiological cardiac repolarization (Rossow et al., 2006) as well as in the generation of an arrhythmogenic substrate in heart failure (Rossow et al., 2004). Since cardiomyocytes dramatically increase their intracellular Ca^2+^ concentration during each heartbeat, an important problem to be solved is the question whether the “contractile Ca^2+^” released by LTCC and RyR2 or a different pool of Ca^2^ regulated by LTCC and/or canonical transient receptor potential channels (TRPC1, 3 or 6) activate PP2B in CVD (Rossow et al., 2004).

### Connexin 43 and SAP97—Reversible Protein Phosphorylation at Cell–Cell Contacts

Cardiac electrical excitation is mainly conducted from cell to cell by connexins. In heart especially connexins 43 and 45 fulfill an important function for conduction and increased phosphatase activity of either PP1 or PP2A was shown to induce conduction slowing possibly due to influence on protein stability and nearby channel activation (Jeyaraman et al., 2003; Ai and Pogwizd, 2005; Ai et al., 2011). Decreased connexin expression and phosphorylation was recently identified as a novel inducer of arrhythmic events (Delmar and Makita, 2012; Remo et al., 2012). Another important protein involved in such cell-cell contacts is synapse-associated protein 97 (SAP97). Numerous studies in brain have suggested CaMKII dependent phosphorylation and PP2B-mediated dephosphorylation of this scaffolding protein (Nikandrova et al., 2010). While SAP97 is also expressed in heart and initial studies in mice have revealed potential impact on potassium channel function and thereby action potential duration, there is a lack of knowledge regarding reversible SAP97 phosphorylation in the heart.

### Glycogen Synthase and Phosphorylase—Reversible Protein Phosphorylation During Cardiac Metabolism

Although being the first PP substrates studied in terms of regulation and mechanism, the knowledge about functional impact of phosphatases on proteins of the glycogen metabolism has been underestimated for cardiac function a long time. However, recent milestone publications have led to a revival of the study of metabolic pathways and their contribution toward CVDs (Kolwicz et al., 2013; Chouchani et al., 2014; Mirtschink et al., 2015). The most important substrates of PP1 and PP2A are glycogen synthase, glycogen phosphorylase and to a lesser extent, phosphorylase kinase (Ceulemans and Bollen, 2004). PP1 and PP2A-mediated dephosphorylation of the rate-limiting enzymes of glycogen metabolism results in the storage of glycogen, in accordance with the proposed function of PP1 as an energy conserving enzyme (Ceulemans and Bollen, 2004). Future studies will be necessary to understand the exact contribution of the different phosphatases in cardiac cells in this process and to translate these findings for the understanding of pathophysiological processes. In this regard it will also be essential to check reversible phosphorylation in other metabolic pathways, e.g., lipid, amino acid, and nucleotide metabolism.

While this chapter indicated the widespread target portfolio of PPs in the heart, the molecular regulation of PPs themselves is at least equally important for cardiac physiology and manifestation of CVDs. Therefore we are going to review the multiple checkpoints of PP regulation in the heart in the light of PP-dependent PK activity regulation.

## Regulating the Regulators: Molecular Control of Protein Phosphatase Activity in the Healthy and Diseased Heart

PPs themselves can be regulated at multiple checkpoints. We will first discuss and highlight some of this regulative mechanisms and subsequently summarize the consequences of deregulated phosphatase activity in CVDs.

The perhaps most obvious but still not really well studied mechanism of transcriptional control is the expression of different isoforms of the catalytic subunits of PP1, PP2A, and PP2B (Cohen, 2002c; Korrodi-Gregorio et al., 2014). Especially for PP1, isoform analysis in non-cardiac cells has shown difference in subcellular distribution of catalytic isoforms and response toward cellular stressors like hypoxia and/or apoptosis (Wang et al., 2001; Comerford et al., 2006; Iacobazzi et al., 2015). The most obvious but however not really well studied mechanism of transcriptional control is the expression of different isoforms of the catalytic subunits of PP1, PP2A, and PP2B (Herzig and Neumann, 2000; Felkin et al., 2011; DeGrande et al., 2013; Korrodi-Gregorio et al., 2014). Especially for PP1, isoform analysis in non-cardiac cells has shown difference in subcellular distribution of catalytic isoforms and response toward cellular stressors like hypoxia and/or apoptosis (Ceulemans and Bollen, 2004; Trinkle-Mulcahy et al., 2006; Djouder et al., 2007; Iacobazzi et al., 2015). Mutants of different PP1 isoforms in *Drosophila* or mice give rise to apparently different phenotypes (Raghavan et al., 2000; Kirchner et al., 2007). For cardiac myocytes there is yet very limited evidence that isoform-specific distribution of PP catalytic subunits underlies functional implication during CVDs, thus awaiting further examination in suitable cellular or animal models. So far preliminary reports suggest predominant expression of PP1α in myofibrillar, PP1β in longitudinal SR and PP1γ in the junctional SR fraction under basal conditions (Aoyama et al., 2011). However, the small number of PP1c isoforms, their near 90% amino-acid sequence identity and their broad and similar substrate specificities *in vitro* support the current hypothesis that it is predominantly the regulatory subunits with which PP1c interacts that controls the specificity and enormous diversity of PP1 function. To our knowledge there is only one report about isoform specific distribution of PP2A or PP2B catalytic subunits in cardiac cells until today enforcing the importance of regulatory subunits for substrate specificity and subcellular distribution also for the two latter phosphatases (DeGrande et al., 2013). In addition there is some limited knowledge about the characteristic of the calcineurin promoter, while we are not aware of any reports about PP1 or PP2A promoter analysis in cardiac cells leaving a huge unexplored research field of PP activity regulation (Oka et al., 2005). Finally some sparse studies hint at epigenetic regulation mechanisms of, e.g., PP2A expression control but again specific and detailed mechanistic studies in cardiac cells are missing (Keen et al., 2004). Thus although deregulated expression of PPs have been reported during a variety of different CVDs, and the underlying mechanistic nature has not been studied in detail yet.

An interesting opportunity of post-transcriptional PP activity control is conducted via regulated expression of microRNAs (miRs). However, most of the known examples in the heart have revealed control of PP1, PP2A, and PP2B regulatory subunit rather than catalytic subunit expression by miRs so far. Prominent examples are, e.g., miR-1 and miR-133 control of RyR2/PP2A mediated arrhythmogenesis, miR-34-a control of PPP1R10 during cardiac aging and contraction, as well as miR-212/132 control of calcineurin activity during cardiac hypertrophy (Belevych et al., 2011; Ucar et al., 2012; Boon et al., 2013). It is anticipated that miR-mediated control of PP catalytic and regulatory subunits may serve as a novel attractive and highly specific pharmacological target among CVDs.

The most intensive studied way of PP activity control is, however, post-translational PP activity control, mediated either by a regulatory or an inhibitory subunit. Recent studies have suggested that the number of PIPs is bigger than the number of PP, leading to a situation of competition between the different regulatory and inhibitory subunits for the catalytic subunits of PPs (Ceulemans and Bollen, 2004). Genetic studies in transgenic mice with knock-out or overexpression of PP regulatory subunits have enforced our understanding about this process (Heijman et al., 2013). While there are, e.g., already more than 150 known regulatory subunits for PP1, we have decided to focus on two complex examples of PP activity regulation that may serve a prototype for PP regulation and inhibition, namely I-1 and spinophilin. For a comprehensive overview we would like to recommend some excellent reviews dedicated to posttranslational PP activity control (Cohen, 2002c; Redden and Dodge-Kafka, 2011).

### Protein Phosphatase Inhibitor-1 (I-1)

One of the best studied examples of PP-inhibitory subunits in the heart is the I-1. Originally identified in rabbit skeletal muscle, it’s expression has now been verified in nearly every tissue with highest expression in skeletal muscle, adipocytes, kidney and liver and brain (Huang and Glinsmann, 1976; Hemmings et al., 1992; Aleem et al., 2001). Interestingly I-1 can only be found in vertebrates and may therefore represent rather recent addition of the fine-tuning interplay between PKA and PP1 from an evolutionary view (Ceulemans and Bollen, 2004). I-1 is one of the few thermostable proteins and cannot be precipitated by 1% trichloroacetic acid (Aitken and Cohen, 1982; El-Armouche et al., 2004; Wittkopper et al., 2011). Although being relatively small and without any transmembrane domains, the crystal structure of I-1 could not be elucidated until recently (Huang et al., 2010). As the exact activation and inhibition mechanism of I-1 has been already been described before in this review we will know focus on the consequence of deregulated I-1 expression on PP activity and *vice versa* for different CVDs.

The first example comes from failing human myocardium, which showed hyperactive PP1 (Neumann et al., 1997; Huang et al., 1999; Schwoerer et al., 2008). It was tempting to speculate that I-1 might be dysregulated in heart failure and in fact I-1 mRNA and total I-1 protein amount were reduced by 60%, and its PKA-dependent phosphorylation level even by 80% (El-Armouche et al., 2003, 2004). The reason of decreased I-1 phosphorylation possibly reflects desensitization of β-AR signaling with decreased cAMP levels and PKA activation in the failing myocardium. Additionally an increase in calcineurin activity, especially in settings of elevated diastolic Ca^2+^ levels, may contribute to the decrease in I-1 phosphorylation described above (Lim and Molkentin, 1999). Finally I-1 protein expression was also markedly downregulated in an experimental dog model of heart failure arguing for a central role of I-1 mediated PP1 activity control during heart failure (Gupta et al., 2003; El-Armouche et al., 2007a). All these data suggest that I-1 downregulation may be part of the desensitization process taking place among the irregular β-adrenergic signal transduction pathway in failing hearts. A hypothesis that could in part be validated by studies using rats with chronic isoprenaline treatment showing decreased I-1 expression and activation levels as well (El-Armouche et al., 2007a). Knock-out of I-1 in mice in two different studies led to either none or a subtle decrease in basal contractile parameters and a significantly blunted β-adrenergic response (Carr et al., 2002; El-Armouche et al., 2008). Isolated atria from I-1 deleted mice showed normal isometric force under basal and maximally stimulated conditions but a rightward shift of the concentration-response curve of isoprenaline (Carr et al., 2002). Importantly, neither the β-AR density nor the PP1 protein levels differed from wild type littermates, indicating no obvious major compensatory changes in the I-1 KO hearts. Finally, germline deletion of I-1 did not result in apparent heart disease and did not negatively affect life expectancy and/or heart structure in aged mice arguing against a causative role of diminished I-1 expression for heart failure progression (Carr et al., 2002; El-Armouche et al., 2008; Heijman et al., 2013). Further studies are needed to better understand the functional implication of I-1 deletion during experimental models of heart failure and to elucidate if pharmacological inhibition of I-1 might be beneficial or detrimental during initiation and progression of heart failure. In contrast extreme overexpression of full-length I-1 (200-fold) led to spontaneous cardiac hypertrophy and cardiac dysfunction accompanied by compensatory PP1 expression already at young age of 3 months (El-Armouche et al., 2008). Instead conditional cardiomyocyte-specific and moderate overexpression of a constitutively active I-1 (I-1c) show a hypercontractile response comparable to PLB knock out mice but exaggerated contractile dysfunction upon catecholamine application in young mice and spontaneous development of dilated cardiomyopathy in adult mice (Wittkopper et al., 2010b, 2011). A comparative PP1 upregulation could however not be detected in this mouse model. Another study did not show any overt cardiac phenotypes in young mice after moderate I-1 overexpression and therefore postulated moderate I-1 overactivation/overexpression as a potential pharmacological approach (Pathak et al., 2005). Indeed subsequent studies using models of pressure overload and chronic isoprenaline infusion-induced heart failure, suggested that I-1 overexpression is associated with a preserved cardiac function, an attenuated development of cardiac hypertrophy and a lower degree of fibrosis and apoptosis (Pathak et al., 2005; Chen et al., 2010; Ishikawa et al., 2014).

A second example of I-1 controlled PP1 activity in a pathological condition has been described for the occurrence and persistence of atrial fibrillation (El-Armouche et al., 2006b; Chiang et al., 2015). Similar to the situation in failing myocardium some proteins which are important for Ca^2+^ handling, such as the L-type Ca^2+^ channel or cMyBP showed increased dephosphorylation hinting at hyperactive PPs (El-Armouche et al., 2006b; Dobrev and Nattel, 2008; Nattel et al., 2008). Nevertheless some other Ca^2+^-handling proteins like the RyR2 (at Ser2814) or PLB (at Thr17) were in turn hyper-phosphorylated arguing for rather subcellular than general upregulation of PP activity (Neef et al., 2010). The exact mechanism for this observation is however still unknown and deserves further investigation. Another contrast to the situation in failing myocardium were the level of I-1 as well as S67-phospho I-1, which were unchanged between AF and non-AF patients (Bhasin et al., 2007). Even more surprisingly, PKA-dependent phosphorylation of I-1 at Thr35 was roughly 10-fold higher in cAF patients, which is thought to be completely suppressive for SR-bound PP1 activity, thus leading to increased phosphorylation of PLB (Ser16) and RyR2 (Ser2814; El-Armouche et al., 2006b; Chelu et al., 2009; Greiser et al., 2009). Another contributor toward RyR2 phosphorylation in cAF patients may be overactivated CaMKII which overcomes the globally enhanced PP1 activity causing greater steady-state RyR2 phosphorylation (Chelu et al., 2009).

As a final example for I-1 controlled PP activity during CVDs we have picked ventricular arrhythmias, which may be responsible for almost half of death cases in patients with heart failure (Packer, 1985; Roberts-Thomson et al., 2011). In general ventricular arrhythmias are initiated and maintained by either focal (ectopic) or re-entry mechanisms, which have been linked to increased diastolic SR Ca^2+^ leak from RyR2 and subsequent activation of the Na^+^/Ca^2+^-exchanger (NCX) at the cellular level (Weiss et al., 2000; Jalife et al., 2003; Prystowsky et al., 2012; Qu and Weiss, 2015). Increased and/or prolonged NCX activity may in turn lead to delayed after-depolarizations underlying arrhythmogenesis (Vermeulen et al., 1994; Schillinger et al., 2003; Sipido et al., 2007). Although still under debate if hyperactivated PKA or CaMKII play the major role for increased RyR2 activation and subsequent SR Ca^2+^ leak, it is common sense that β-adrenoceptor stimulation is the general trigger for SR Ca^2+^ leak in both atrial and ventricular arrhythmias (Eisner et al., 2009; Eschenhagen, 2010; Dobrev et al., 2011). In contrast much less is known about the physiological and pathological role of RyR2 dephosphorylation by phosphatases. Different studies have shown that both, PP1 and PP2A are coupled to the RyR2 macromolecular complex (Marx et al., 2000). Consequently, the dual PP1/PP2A inhibitor okadaic acid increases the RyR2 open probability and phosphorylated RyR2 subunits have been shown to be dephosphorylated using recombinant PP1 (Sonnleitner et al., 1997).

Interestingly, I-1 deleted mice were partly protected from structural (hypertrophy, fibrosis, and dilatation) and functional effects (loss of inotropic response to dobutamine) of chronic β-adrenoceptor stimulation via isoprenaline infusions with minipumps (Wittkopper et al., 2011). In addition, injections of increasing doses of isoprenaline with continued ECG monitoring showed marked protection from fatal catecholamine induced arrhythmias in I-1 knock-out mice (El-Armouche et al., 2008). Notably, the protection from β-adrenergic-mediated cardiotoxicity in I-1 knock-out mice was not associated with changes in heart rate regulation. Further studies using, e.g., isoform-specific PP1 and/or PP2A overexpressing mice or cardiac myocytes may help to strengthen these preliminary results.

### Spinophilin

Another fascinating example of phosphatase activity regulation with potential implication in CVD can be found within the interaction of spinophilin and PP1. First evidence that spinophilin can act as a regulatory PP1 subunits stems from *in vitro* co-immunoprecipitation studies using overexpressed GST-RyR2 fusion proteins (Marx et al., 2001). While *in vitro* co-incubation of spinophilin and PP1 with the model substrate phosphorylase A led to decreased dephosphorylation activity, *in vivo* experiments showed that spinophilin acts as a scaffolding protein to target PP1 toward its endogenous substrates, thus rather facilitating dephosphorylation activity than decreasing it (Feng et al., 2000; Oliver et al., 2002). Elegant studies using different PP1/spinophilin or other PP1/PIP crystal complexes helped to understand the underlying mechanistic principles of these interactions (Ragusa et al., 2010, 2011).

It was shown that the C-terminal domain (aa 417–494) of spinophilin is necessary and sufficient for complete interaction with PP1 (Hsieh-Wilson et al., 1999). However, crystallization studies revealed that the PP1-binding domain of spinophilin is highly unstructured and has contact with multiple residues on PP1. In fact, the spinophilin binding domain entirely folds only upon binding to PP1 (Ragusa et al., 2010). Interestingly, spinophilin does not only bind via the well-recognized RVXF binding motif, but also by multiple interaction with the C-terminal groove of PP1. Most importantly, despite all the above described PP1/spinophilin interaction sites, spinophilin does not affect the PP1 active site, nor two further putative substrate binding sites, enabling full PP1 activity when bound to spinophilin and leaving the chance for further PP1/PIP interactions. As a consequence really unique PP1 holoenzymes can exist, which may allow for very specific counteracting and fine-tuning of phosphorylation events conducted by the plethora of specific kinases within the cell. It would be of major interest to solve the structure of PP1/I-1 complexes as well, to better understand the nature of the so far mainly phenomenologically described interaction/inhibition.

Further reports showed that spinophilin is able to direct PP1 to the membrane of the SR in cardiac myocytes. Cardiomyocytes from spinophilin knock out mice revealed significantly reduced length, increased Ca^2+^ amplitude as well as maximal rate of Ca^2+^ rise during systole, decreased shortening amplitude and maximal rate of shortening, while β-adrenergic stimulation remained intact (Petzhold et al., 2011). Mechanistic studies suggested that spinophilin-mediated PP1 targeting to RyR2 and Phospholamban lead to site-specific enhanced dephosphorylation, thus evoking the phenotypic consequences described in the latter study. Very recently the group around Xander Wehrens transferred this knowledge into better understanding of disease mechanisms in atrial fibrillation putatively underlying deregulated PP1/spinophilin interactions and subsequent hyperactivation of RyR2 (Chiang et al., 2014). In detail they found that knockout of sphinophilin-1 resulted in strongly reduced interaction of PP1 and RyR2, while RyR2 phosphorylation was significantly increased at serine (Ser) 2814 but unchanged at Ser2808. As a result RyR2s from spinophilin-1 KO mice showed the expected increase in RyR2 open probability in lipid bilayer experiments, increased Ca^2+^ spark frequency in isolated atrial myocytes, which could be reverted by CaMKII inhibition, and most importantly increased atrial ectopy and susceptibility to pacing-induced atrial fibrillation *in vivo*, which could be reverted by crossing in phosphorylation-dead RyR2-Ser2814A mice (Chiang et al., 2014). In summary this reports showed the first time that decreased local PP1 regulation of RyR2 contributes to RyR2 hyperactivity and promotion of atrial fibrillation susceptibility. This milestone study may set the basis for more exact examination of localized PP activity in CVDs, thus enabling novel drug targeting strategies in the future.

The last chapter of PP-dependent PK activity regulation will be opened in the upcoming section of this review. Hereby direct activity control of PKs via PP-mediated dephosphorylation will be discussed in light of a rapid molecular mechanism to achieve direct feedback control of PK activity.

## Direct Control of Protein Kinase Expression and Activity by Protein Phosphatases

It is a basic principle that PKs themselves are activated by (auto-)phosphorylation (Ubersax and Ferrell, 2007). In order to avoid excessive PK activation it is necessary that in most cases PK phosphorylation has to be reversible. Indeed several examples (see also Table 1) show direct PP-mediated dephosphorylation of PKs in cardiac cells. However, PP-mediated dephosphorylation of two of the most abundant and essential PKs in cardiac myocytes, namely PKA and CaMKII has only be studied in non-cardiac tissues until today (Blitzer et al., 1998; Bradshaw et al., 2003; Humphries et al., 2005; Otmakhov et al., 2015).

Dephosphorylation of PKA at position Thr-197 by PP2A was shown *in vitro* and *in vivo*. Interestingly, dephosphorylation activity strongly depends on oxidative state, which is mechanistically mediated via the neighboring cysteine 199 (Humphries et al., 2005). CaMKII is auto-activated at position Thr286 and can be dephosphorylated by PP1 and PP2B (Reese et al., 2011). In this case the dephosphorylation activity in dendritic spines is calcium dependent. It is tempting to speculate that PKA as well as CaMKII are also direct substrates of PP-mediated dephosphorylation in cardiac physiology and especially in CVDs, thus predisposing further studies to clarify this issue.

In addition, there is yet another possibility of more or less direct control of PK activity by PP mediated initiation of alternative splicing in cardiac myocytes. The best studied example in this case is the CaMKIIδ-isoform, which plays an important role during evolvement of heart failure and cardiac arrhythmias (Gray and Heller Brown, 2014). Over- or deregulated expression of single splicing variants of CaMKIIδ are sufficient to induce cardiac hypertrophy, dilated cardiomyopathy and subsequent heart failure in experimental animal models (Erickson, 2014). Two studies have shown that reversible phosphorylation of the splicing factor SF2/ASF by PKA and PP1 is able to change the splicing pattern of CaMKIIδ and subsequently trigger functional consequences on typical CaMKII substrates such as PLB (Gu et al., 2011; Huang et al., 2014).

As described before Histones, Histone Deacetylases as well as transcription and translation factors have also been identified as PP substrates (Backs et al., 2006; Heijman et al., 2013; Kreusser and Backs, 2014). All these components are necessary prerequisites for (epi-)genetic control of PK activity. Finally, as CaMKIIδ is by far not the only cardiac PK undergoing alternative splicing it will be worth to examine this novel way of PP-mediated PK regulation in more detail in the future. Regulation of alternative splicing in cardiac myocytes has emerged as a highly innovative and novel drug target in multiple studies and would be interesting to better understand the contribution of PPs in this regard. This conclusion closes the gap toward the final chapter of this review, which discusses the current state-of-the-art knowledge regarding pharmacological PP activity control that may serve as an additional or synergistic therapeutic approach toward pharmacological compounds targeting PKs.

## Therapeutic Perspective

The widespread tasks of PP1, PP2A, and PP2B in healthy and diseased described in the chapter above, predispose the PPs as novel drug targets. In order to receive similar attention as kinase inhibitors, there are however still some challenges that have to be tackled.

The most straightforward approach to block the activity of an enzyme is the design of small molecules (SMOLs) which bind to the active site of the enzyme, thus blocking access for their endogenous substrates. Due to the very high structural conservation in PP1’s catalytic site with that of PP2A, it has remained difficult to specifically target the active site of either phosphatase (Terrak et al., 2004; Cho and Xu, 2007). Nevertheless, some natural toxins such as calyculin, cantharidin, tautomycin and okadaic acid did reveal subtle selectivity between PP1 and PP2A *in vitro* and have thus been employed the last decades to study the role of PP1 and PP2A in cellular signaling processes (Hescheler et al., 1988; Aggen et al., 1999; Fagerholm et al., 2010). Even better selectivity is achieved by the endogenous inhibitors of PP1 and PP2A. As an example the heat stable endogenous protein inhibitors Inhibitor-1 as well as Inhibitor-2 specifically inhibit PP1 at very low nanomolar concentrations without affecting the any other known serine/threonine phosphatase (Kwon et al., 1997). The usefulness of the latter inhibitors in functional assays or as drug leads is limited by the size, availability, stability as well as the cellular permeability of these proteins, but they could nevertheless be used as starting points for peptide-based drug design (Chatterjee and Kohn, 2013). Due to the multitude of phosphatase substrates direct and solely targeting of a single phosphatase catalytic subunit will putatively be insufficient or induce serious toxic side effects so further strategies rely on either disrupting the interaction between (a) the catalytic and respective regulatory subunit or (b) the regulatory subunit and the respective substrate. As a proof-of-concept specific modulators of PP1 (and PP2A) holoenzymes have been developed not only for the treatment of CVDs but also for the treatment of diabetes, Parkinson’s disease and drug addiction (Uehata et al., 1997; Armstrong et al., 1998; McConnell and Wadzinski, 2009; Yger and Girault, 2011). Concrete examples are disruption of the PP1/GADD34 complex by salubrinal or guanabenz or I-1/I-2 overexpression in rodent/pig models of myocardial infarction (Boyce et al., 2005; Tsaytler et al., 2011; Liu et al., 2012; Neuber et al., 2014). Moreover synthetic peptides containing the PP1c-binding motif (RVxF) have been successfully used to disrupt PP1 complexes in a neuronal and cardiac context, inducing beneficial effects on synaptic transmission and PLB phosphorylation (Reither et al., 2013; Sotoud et al., 2015). Thus, clinical targeting of PPs side-by-side with PK inhibition may represent a promising opportunity to treat CVDs in the near future.

Last but not least we propose two novel strategies to further enhance the feasibility of PP targeting drugs in CVDs: 1. Engaging subcellular and isotype-specific control of PPs: a recent review has highlighted the isotype-specific interaction of PP1 with different regulatory subunits and inhibitors. Combined with compartment-specific PP1 targeting this knowledge might lead to the development of really specific PP targeting drugs. 2. Combination of PK inhibitor and Phosphatase activating drugs and *vice versa*: in order to increase potency of currently used drugs, exact knowledge about the contribution of kinases and phosphatases during reversible protein phosphorylation of a specific substrate may help to develop novel and successful combination therapies—similar to the success story of LCZ-696/Entresto (Gu et al., 2010; Solomon et al., 2012; Jessup et al., 2014; Desai et al., 2015).

### Conflict of Interest Statement

The authors declare that the research was conducted in the absence of any commercial or financial relationships that could be construed as a potential conflict of interest.

## References

[B1] Abiria S. A. Colbran R. J. (2010). CaMKII associates with CaV1.2 L-type calcium channels via selected β subunits to enhance regulatory phosphorylation. J. Neurochem. 112, 150–161. 10.1111/j.1471-4159.2009.06436.x 19840220 PMC2814318

[B2] Aggen J. B. Humphrey J. M. Gauss C. M. Huang H. B. Nairn A. C. Chamberlin A. R. (1999). The design, synthesis, and biological evaluation of analogues of the serine-threonine protein phosphatase 1 and 2A selective inhibitor microcystin LA: rational modifications imparting PP1 selectivity. Bioorg. Med. Chem. 7, 543–564. 10.1016/S0968-0896(98)00254-5 10220039

[B3] Aitken A. Cohen P. (1982). Isolation and characterisation of active fragments of protein phosphatase inhibitor-1 from rabbit skeletal muscle. FEBS Lett. 147, 54–58. 10.1016/0014-5793(82)81010-7 7140990

[B4] Ai X. Jiang A. Ke Y. Solaro R. J. Pogwizd S. M. (2011). Enhanced activation of p21-activated kinase 1 in heart failure contributes to dephosphorylation of connexin 43. Cardiovasc. Res. 92, 106–114. 10.1093/cvr/cvr163 21727092 PMC3172982

[B5] Ai X. Pogwizd S. M. (2005). Connexin 43 downregulation and dephosphorylation in nonischemic heart failure is associated with enhanced colocalized protein phosphatase type 2A. Circ. Res. 96, 54–63. 10.1161/01.RES.0000152325.07495.5a 15576650

[B6] Aleem E. A. Flohr T. Hunziker A. Mayer D. Bannasch P. Thielmann H. W. (2001). Detection and quantification of protein phosphatase inhibitor-1 gene expression in total rat liver and isolated hepatocytes. Mol. Cell. Biochem. 217, 1–12. 10.1023/A:1007141514750 11269652

[B7] Aoyama H. Ikeda Y. Miyazaki Y. Yoshimura K. Nishino S. Yamamoto T. (2011). Isoform-specific roles of protein phosphatase 1 catalytic subunits in sarcoplasmic reticulum-mediated Ca^2+^ cycling. Cardiovasc. Res. 89, 79–88. 10.1093/cvr/cvq252 20675715

[B8] Armstrong C. G. Doherty M. J. Cohen P. T. (1998). Identification of the separate domains in the hepatic glycogen-targeting subunit of protein phosphatase 1 that interact with phosphorylase a, glycogen and protein phosphatase 1. Biochem. J. 336, 699–704. 10.1042/bj3360699 9841883 PMC1219922

[B9] Ashpole N. M. Herren A. W. Ginsburg K. S. Brogan J. D. Johnson D. E. Cummins T. R. (2012). Ca^2+^/calmodulin-dependent protein kinase II (CaMKII) regulates cardiac sodium channel NaV1.5 gating by multiple phosphorylation sites. J. Biol. Chem. 287, 19856–19869. 10.1074/jbc.M111.322537 22514276 PMC3370170

[B10] Auger-Messier M. Accornero F. Goonasekera S. A. Bueno O. F. Lorenz J. N. Van Berlo J. H. (2013). Unrestrained p38 MAPK activation in Dusp1/4 double-null mice induces cardiomyopathy. Circ. Res. 112, 48–56. 10.1161/CIRCRESAHA.112.272963 22993413 PMC5929162

[B11] Awad S. Kunhi M. Little G. H. Bai Y. An W. Bers D. (2013). Nuclear CaMKII enhances histone H3 phosphorylation and remodels chromatin during cardiac hypertrophy. Nucleic Acids Res. 41, 7656–7672. 10.1093/nar/gkt500 23804765 PMC3763528

[B12] Aye T. T. Soni S. Van Veen T. A. Van Der Heyden M. A. Cappadona S. Varro A. (2012). Reorganized PKA-AKAP associations in the failing human heart. J. Mol. Cell Cardiol. 52, 511–518. 10.1016/j.yjmcc.2011.06.003 21712045

[B13] Baba S. Dun W. Boyden P. A. (2004). Can PKA activators rescue Na^+^ channel function in epicardial border zone cells that survive in the infarcted canine heart? Cardiovasc. Res. 64, 260–267. 10.1016/j.cardiores.2004.06.021 15485685

[B14] Backs J. Song K. Bezprozvannaya S. Chang S. Olson E. N. (2006). CaM kinase II selectively signals to histone deacetylase 4 during cardiomyocyte hypertrophy. J. Clin. Invest. 116, 1853–1864. 10.1172/JCI27438 16767219 PMC1474817

[B15] Barefield D. Sadayappan S. (2010). Phosphorylation and function of cardiac myosin binding protein-C in health and disease. J. Mol. Cell Cardiol. 48, 866–875. 10.1016/j.yjmcc.2009.11.014 19962384 PMC6800196

[B16] Beals C. R. Sheridan C. M. Turck C. W. Gardner P. Crabtree G. R. (1997). Nuclear export of NF-ATc enhanced by glycogen synthase kinase-3. Science 275, 1930–1934. 10.1126/science.275.5308.1930 9072970

[B17] Belevych A. E. Sansom S. E. Terentyeva R. Ho H. T. Nishijima Y. Martin M. M. (2011). MicroRNA-1 and -133 increase arrhythmogenesis in heart failure by dissociating phosphatase activity from RyR2 complex. PLoS ONE 6:e28324. 10.1371/journal.pone.0028324 22163007 PMC3232211

[B18] Belmonte S. L. Blaxall B. C. (2011). G protein coupled receptor kinases as therapeutic targets in cardiovascular disease. Circ. Res. 109, 309–319. 10.1161/CIRCRESAHA.110.231233 21778435 PMC3146028

[B19] Berrebi-Bertrand I. Souchet M. Camelin J. C. Laville M. P. Calmels T. Bril A. (1998). Biophysical interaction between phospholamban and protein phosphatase 1 regulatory subunit GM. FEBS Lett. 439, 224–230. 10.1016/S0014-5793(98)01364-7 9845327

[B20] Bers D. M. Herren A. W. (2012). Na^+^ channel I-II loop mediates parallel genetic and phosphorylation-dependent gating changes. Circulation 126, 2042–2046. 10.1161/CIRCULATIONAHA.112.140384 23091083 PMC3502635

[B21] Bettoun D. J. Buck D. W. II Lu J. Khalifa B. Chin W. W. Nagpal S. (2002). A vitamin D receptor-Ser/Thr phosphatase-p70 S6 kinase complex and modulation of its enzymatic activities by the ligand. J. Biol. Chem. 277, 24847–24850. 10.1074/jbc.C200187200 12036952

[B22] Bhasin N. Cunha S. R. Mudannayake M. Gigena M. S. Rogers T. B. Mohler P. J. (2007). Molecular basis for PP2A regulatory subunit B56α targeting in cardiomyocytes. Am. J. Physiol. Heart Circ. Physiol. 293, H109–H119. 10.1152/ajpheart.00059.2007 17416611

[B23] Blaich A. Welling A. Fischer S. Wegener J. W. Kostner K. Hofmann F. (2010). Facilitation of murine cardiac L-type Ca(v)1.2 channel is modulated by calmodulin kinase II-dependent phosphorylation of S1512 and S1570. Proc. Natl. Acad. Sci. U.S.A. 107, 10285–10289. 10.1073/pnas.0914287107 20479240 PMC2890469

[B24] Blitzer R. D. Connor J. H. Brown G. P. Wong T. Shenolikar S. Iyengar R. (1998). Gating of CaMKII by cAMP-regulated protein phosphatase activity during LTP. Science 280, 1940–1942. 10.1126/science.280.5371.1940 9632393

[B25] Bollen M. Beullens M. (2002). Signaling by protein phosphatases in the nucleus. Trends Cell Biol. 12, 138–145. 10.1016/S0962-8924(01)02247-4 11859026

[B26] Boon R. A. Iekushi K. Lechner S. Seeger T. Fischer A. Heydt S. (2013). MicroRNA-34a regulates cardiac ageing and function. Nature 495, 107–110. 10.1038/nature11919 23426265

[B27] Bourajjaj M. Armand A. S. Da Costa Martins P. A. Weijts B. Van Der Nagel R. Heeneman S. (2008). NFATc2 is a necessary mediator of calcineurin-dependent cardiac hypertrophy and heart failure. J. Biol. Chem. 283, 22295–22303. 10.1074/jbc.M801296200 18477567

[B28] Boyce M. Bryant K. F. Jousse C. Long K. Harding H. P. Scheuner D. (2005). A selective inhibitor of eIF2 dephosphorylation protects cells from ER stress. Science 307, 935–939. 10.1126/science.1101902 15705855

[B29] Bradshaw J. M. Kubota Y. Meyer T. Schulman H. (2003). An ultrasensitive Ca^2+^/calmodulin-dependent protein kinase II-protein phosphatase 1 switch facilitates specificity in postsynaptic calcium signaling. Proc. Natl. Acad. Sci. U.S.A. 100, 10512–10517. 10.1073/pnas.1932759100 12928489 PMC193592

[B30] Brandl C. J. Green N. M. Korczak B. Maclennan D. H. (1986). Two Ca^2+^ ATPase genes: homologies and mechanistic implications of deduced amino acid sequences. Cell 44, 597–607. 10.1016/0092-8674(86)90269-2 2936465

[B31] Brandmayr J. Poomvanicha M. Domes K. Ding J. Blaich A. Wegener J. W. (2012). Deletion of the C-terminal phosphorylation sites in the cardiac β-subunit does not affect the basic β-adrenergic response of the heart and the Ca(v)1.2 channel. J. Biol. Chem. 287, 22584–22592. 10.1074/jbc.M112.366484 22589548 PMC3391128

[B32] Braz J. C. Gregory K. Pathak A. Zhao W. Sahin B. Klevitsky R. (2004). PKC- regulates cardiac contractility and propensity toward heart failure. Nat. Med. 10, 248–254. 10.1038/nm1000 14966518

[B33] Brinkworth R. I. Breinl R. A. Kobe B. (2003). Structural basis and prediction of substrate specificity in protein serine/threonine kinases. Proc. Natl. Acad. Sci. U.S.A. 100, 74–79. 10.1073/pnas.0134224100 12502784 PMC140887

[B34] Carlisle Michel J. J. Dodge K. L. Wong W. Mayer N. C. Langeberg L. K. Scott J. D. (2004). PKA-phosphorylation of PDE4D3 facilitates recruitment of the mAKAP signalling complex. Biochem. J. 381, 587–592. 10.1042/BJ20040846 15182229 PMC1133866

[B35] Carr A. N. Schmidt A. G. Suzuki Y. Del Monte F. Sato Y. Lanner C. (2002). Type 1 phosphatase, a negative regulator of cardiac function. Mol. Cell. Biol. 22, 4124–4135. 10.1128/MCB.22.12.4124-4135.2002 12024026 PMC133876

[B36] Ceulemans H. Bollen M. (2004). Functional diversity of protein phosphatase-1, a cellular economizer and reset button. Physiol. Rev. 84, 1–39. 10.1152/physrev.00013.2003 14715909

[B37] Chatterjee J. Kohn M. (2013). Targeting the untargetable: recent advances in the selective chemical modulation of protein phosphatase-1 activity. Curr. Opin. Chem. Biol. 17, 361–368. 10.1016/j.cbpa.2013.04.008 23647984

[B38] Chelu M. G. Sarma S. Sood S. Wang S. Van Oort R. J. Skapura D. G. (2009). Calmodulin kinase II-mediated sarcoplasmic reticulum Ca^2+^ leak promotes atrial fibrillation in mice. J. Clin. Invest. 119, 1940–1951. 10.1172/jci37059 19603549 PMC2701862

[B39] Chemin J. Mezghrani A. Bidaud I. Dupasquier S. Marger F. Barrere C. (2007). Temperature-dependent modulation of CaV3 T-type calcium channels by protein kinases C and A in mammalian cells. J. Biol. Chem. 282, 32710–32718. 10.1074/jbc.M702746200 17855364

[B40] Chen E. S. Zhang K. Nicolas E. Cam H. P. Zofall M. Grewal S. I. (2008). Cell cycle control of centromeric repeat transcription and heterochromatin assembly. Nature 451, 734–737. 10.1038/nature06561 18216783

[B41] Chen G. Zhou X. Florea S. Qian J. Cai W. Zhang Z. (2010). Expression of active protein phosphatase 1 inhibitor-1 attenuates chronic β-agonist-induced cardiac apoptosis. Basic Res. Cardiol. 105, 573–581. 10.1007/s00395-010-0106-3 20512582 PMC3095219

[B42] Chen X. Piacentino V. III Furukawa S. Goldman B. Margulies K. B. Houser S. R. (2002). L-type Ca^2+^ channel density and regulation are altered in failing human ventricular myocytes and recover after support with mechanical assist devices. Circ. Res. 91, 517–524. 10.1161/01.RES.0000033988.13062.7C 12242270

[B43] Cheung J. Y. Zhang X. Q. Song J. Gao E. Rabinowitz J. E. Chan T. O. (2010). Phospholemman: a novel cardiac stress protein. Clin. Transl. Sci. 3, 189–196. 10.1111/j.1752-8062.2010.00213.x 20718822 PMC3013348

[B44] Chiang D. Y. Lebesgue N. Beavers D. L. Alsina K. M. Damen J. M. Voigt N. (2015). Alterations in the interactome of serine/threonine protein phosphatase type-1 in atrial fibrillation patients. J. Am. Coll. Cardiol. 65, 163–173. 10.1016/j.jacc.2014.10.042 25593058 PMC4690213

[B45] Chiang D. Y. Li N. Wang Q. Alsina K. M. Quick A. P. Reynolds J. O. (2014). Impaired local regulation of ryanodine receptor type 2 by protein phosphatase 1 promotes atrial fibrillation. Cardiovasc. Res. 103, 178–187. 10.1093/cvr/cvu123 24812280 PMC4133595

[B46] Cho U. S. Xu W. (2007). Crystal structure of a protein phosphatase 2A heterotrimeric holoenzyme. Nature 445, 53–57. 10.1038/nature05351 17086192

[B47] Chouchani E. T. Pell V. R. Gaude E. Aksentijevic D. Sundier S. Y. Robb E. L. (2014). Ischaemic accumulation of succinate controls reperfusion injury through mitochondrial ROS. Nature 515, 431–435. 10.1038/nature13909 25383517 PMC4255242

[B48] Chow C. W. Davis R. J. (2000). Integration of calcium and cyclic AMP signaling pathways by 14-3-3. Mol. Cell. Biol. 20, 702–712. 10.1128/MCB.20.2.702-712.2000 10611249 PMC85175

[B49] Chow C. W. Dong C. Flavell R. A. Davis R. J. (2000). c-Jun NH_2_-terminal kinase inhibits targeting of the protein phosphatase calcineurin to NFATc1. Mol. Cell. Biol. 20, 5227–5234. 10.1128/MCB.20.14.5227-5234.2000 10866678 PMC85971

[B50] Chow C. W. Rincon M. Cavanagh J. Dickens M. Davis R. J. (1997). Nuclear accumulation of NFAT4 opposed by the JNK signal transduction pathway. Science 278, 1638–1641. 10.1126/science.278.5343.1638 9374467

[B51] Chu G. Kranias E. G. (2002). Functional interplay between dual site phospholamban phosphorylation: insights from genetically altered mouse models. Basic Res. Cardiol. 97(Suppl. 1), I43–48. 10.1007/s003950200028 12479233

[B52] Cohen P. (1989). The structure and regulation of protein phosphatases. Annu. Rev. Biochem. 58, 453–508. 10.1146/annurev.bi.58.070189.002321 2549856

[B53] Cohen P. (2002a). The origins of protein phosphorylation. Nat. Cell Biol. 4, E127–E130. 10.1038/ncb0502-e127 11988757

[B54] Cohen P. (2002b). Protein kinases–the major drug targets of the twenty-first century? Nat. Rev. Drug. Discov. 1, 309–315. 10.1038/nrd773 12120282

[B55] Cohen P. T. (2002c). Protein phosphatase 1–targeted in many directions. J. Cell Sci. 115, 241–256. 11839776 10.1242/jcs.115.2.241

[B56] Cole H. A. Perry S. V. (1975). The phosphorylation of troponin I from cardiac muscle. Biochem. J. 149, 525–533. 10.1042/bj1490525 173290 PMC1165658

[B57] Comerford K. M. Leonard M. O. Cummins E. P. Fitzgerald K. T. Beullens M. Bollen M. (2006). Regulation of protein phosphatase 1γ activity in hypoxia through increased interaction with NIPP1: implications for cellular metabolism. J. Cell. Physiol. 209, 211–218. 10.1002/jcp.20726 16826568

[B58] Cori C. F. Schmidt G. Cori G. T. (1939). The synthesis of a polysaccharide from glucose-1-phosphate in muscle extract. Science 89, 464–465. 10.1126/science.89.2316.464 17731092

[B59] Davare M. A. Horne M. C. Hell J. W. (2000). Protein phosphatase 2A is associated with class C L-type calcium channels (Cav1.2) and antagonizes channel phosphorylation by cAMP-dependent protein kinase. J. Biol. Chem. 275, 39710–39717. 10.1074/jbc.M005462200 10984483

[B60] De Arcangelis V. Soto D. Xiang Y. (2008). Phosphodiesterase 4 and phosphatase 2A differentially regulate cAMP/protein kinase a signaling for cardiac myocyte contraction under stimulation of β1 adrenergic receptor. Mol. Pharmacol. 74, 1453–1462. 10.1124/mol.108.049718 18703669 PMC2654611

[B61] DeGrande S. T. Little S. C. Nixon D. J. Wright P. Snyder J. Dun W. (2013). Molecular mechanisms underlying cardiac protein phosphatase 2A regulation in heart. J. Biol. Chem. 288, 1032–1046. 10.1074/jbc.M112.426957 23204520 PMC3542989

[B62] Delmar M. Makita N. (2012). Cardiac connexins, mutations and arrhythmias. Curr. Opin. Cardiol. 27, 236–241. 10.1097/HCO.0b013e328352220e 22382502

[B63] De Munter S. Kohn M. Bollen M. (2013). Challenges and opportunities in the development of protein phosphatase-directed therapeutics. ACS Chem. Biol. 8, 36–45. 10.1021/cb300597g 23214403

[B64] Desai A. S. Mcmurray J. J. Packer M. Swedberg K. Rouleau J. L. Chen F. (2015). Effect of the angiotensin-receptor-neprilysin inhibitor LCZ696 compared with enalapril on mode of death in heart failure patients. Eur. Heart J. 36, 1990–1997. 10.1093/eurheartj/ehv186 26022006

[B65] Djouder N. Metzler S. C. Schmidt A. Wirbelauer C. Gstaiger M. Aebersold R. (2007). S6K1-mediated disassembly of mitochondrial URI/PP1γ complexes activates a negative feedback program that counters S6K1 survival signaling. Mol. Cell. 28, 28–40. 10.1016/j.molcel.2007.08.010 17936702

[B66] Dobrev D. Nattel S. (2008). Calcium handling abnormalities in atrial fibrillation as a target for innovative therapeutics. J. Cardiovasc. Pharmacol. 52, 293–299. 10.1097/FJC.0b013e318171924d 18791467

[B67] Dobrev D. Voigt N. Wehrens X. H. (2011). The ryanodine receptor channel as a molecular motif in atrial fibrillation: pathophysiological and therapeutic implications. Cardiovasc. Res. 89, 734–743. 10.1093/cvr/cvq324 20943673 PMC3039246

[B68] Dodge-Kafka K. L. Bauman A. Mayer N. Henson E. Heredia L. Ahn J. (2010). cAMP-stimulated protein phosphatase 2A activity associated with muscle A kinase-anchoring protein (mAKAP) signaling complexes inhibits the phosphorylation and activity of the cAMP-specific phosphodiesterase PDE4D3. J. Biol. Chem. 285, 11078–11086. 10.1074/jbc.M109.034868 20106966 PMC2856983

[B69] Dodge-Kafka K. L. Langeberg L. Scott J. D. (2006). Compartmentation of cyclic nucleotide signaling in the heart: the role of A-kinase anchoring proteins. Circ. Res. 98, 993–1001. 10.1161/01.RES.0000218273.91741.30 16645149

[B70] Domes K. Ding J. Lemke T. Blaich A. Wegener J. W. Brandmayr J. (2011). Truncation of murine CaV1.2 at Asp-1904 results in heart failure after birth. J. Biol. Chem. 286, 33863–33871. 10.1074/jbc.M111.252312 21832054 PMC3190806

[B71] Egloff M. P. Johnson D. F. Moorhead G. Cohen P. T. Cohen P. Barford D. (1997). Structural basis for the recognition of regulatory subunits by the catalytic subunit of protein phosphatase 1. EMBO J. 16, 1876–1887. 10.1093/emboj/16.8.1876 9155014 PMC1169791

[B72] Eisner D. A. Kashimura T. O’neill S. C. Venetucci L. A. Trafford A. W. (2009). What role does modulation of the ryanodine receptor play in cardiac inotropy and arrhythmogenesis? J. Mol. Cell. Cardiol. 46, 474–481. 10.1016/j.yjmcc.2008.12.005 19150449

[B73] El-Armouche A. Bednorz A. Pamminger T. Ditz D. Didie M. Dobrev D. (2006a). Role of calcineurin and protein phosphatase-2A in the regulation of phosphatase inhibitor-1 in cardiac myocytes. Biochem. Biophys. Res. Commun. 346, 700–706. 10.1016/j.bbrc.2006.05.182 16774736

[B74] El-Armouche A. Boknik P. Eschenhagen T. Carrier L. Knaut M. Ravens U. (2006b). Molecular determinants of altered Ca^2+^ handling in human chronic atrial fibrillation. Circulation 114, 670–680. 10.1161/CIRCULATIONAHA.106.636845 16894034

[B75] El-Armouche A. Eschenhagen T. (2009). β-adrenergic stimulation and myocardial function in the failing heart. Heart Fail. Rev. 14, 225–241. 10.1007/s10741-008-9132-8 19110970

[B76] El-Armouche A. Gocht F. Jaeckel E. Wittkopper K. Peeck M. Eschenhagen T. (2007a). Long-term β-adrenergic stimulation leads to downregulation of protein phosphatase inhibitor-1 in the heart. Eur. J. Heart Fail. 9, 1077–1080. 10.1016/j.ejheart.2007.09.006 17921049

[B77] El-Armouche A. Pohlmann L. Schlossarek S. Starbatty J. Yeh Y. H. Nattel S. (2007b). Decreased phosphorylation levels of cardiac myosin-binding protein-C in human and experimental heart failure. J. Mol. Cell Cardiol. 43, 223–229. 10.1016/j.yjmcc.2007.05.003 17560599

[B78] El-Armouche A. Pamminger T. Ditz D. Zolk O. Eschenhagen T. (2004). Decreased protein and phosphorylation level of the protein phosphatase inhibitor-1 in failing human hearts. Cardiovasc. Res. 61, 87–93. 10.1016/j.cardiores.2003.11.005 14732205

[B79] El-Armouche A. Rau T. Zolk O. Ditz D. Pamminger T. Zimmermann W. H. (2003). Evidence for protein phosphatase inhibitor-1 playing an amplifier role in β-adrenergic signaling in cardiac myocytes. FASEB J. 17, 437–439. 10.1096/fj.02-0057fje 12514122

[B80] El-Armouche A. Wittkopper K. Degenhardt F. Weinberger F. Didie M. Melnychenko I. (2008). Phosphatase inhibitor-1-deficient mice are protected from catecholamine-induced arrhythmias and myocardial hypertrophy. Cardiovasc. Res. 80, 396–406. 10.1093/cvr/cvn208 18689792

[B81] El-Armouche A. Wittkopper K. Fuller W. Howie J. Shattock M. J. Pavlovic D. (2011). Phospholemman-dependent regulation of the cardiac Na/K-ATPase activity is modulated by inhibitor-1 sensitive type-1 phosphatase. FASEB J. 25, 4467–4475. 10.1096/fj.11-184903 21849407

[B82] Endo S. Zhou X. Connor J. Wang B. Shenolikar S. (1996). Multiple structural elements define the specificity of recombinant human inhibitor-1 as a protein phosphatase-1 inhibitor. Biochemistry 35, 5220–5228. 10.1021/bi952940f 8611507

[B83] Erickson J. R. (2014). Mechanisms of CaMKII Activation in the Heart. Front. Pharmacol. 5:59. 10.3389/fphar.2014.00059 24765077 PMC3980116

[B84] Eschenhagen T. (2010). Is ryanodine receptor phosphorylation key to the fight or flight response and heart failure? J. Clin. Invest. 120, 4197–4203. 10.1172/JCI45251 21099119 PMC2994341

[B85] Fagerholm A. E. Habrant D. Koskinen A. M. (2010). Calyculins and related marine natural products as serine-threonine protein phosphatase PP1 and PP2A inhibitors and total syntheses of calyculin A, B, and C. Mar. Drugs 8, 122–172. 10.3390/md80100122 20161975 PMC2817927

[B86] Felkin L. E. Narita T. Germack R. Shintani Y. Takahashi K. Sarathchandra P. (2011). Calcineurin splicing variant calcineurin Aβ1 improves cardiac function after myocardial infarction without inducing hypertrophy. Circulation 123, 2838–2847. 10.1161/CIRCULATIONAHA.110.012211 21632490

[B87] Feng J. Yan Z. Ferreira A. Tomizawa K. Liauw J. A. Zhuo M. (2000). Spinophilin regulates the formation and function of dendritic spines. Proc. Natl. Acad. Sci. U.S.A. 97, 9287–9292. 10.1073/pnas.97.16.9287 10922077 PMC16860

[B88] Feschenko M. S. Sweadner K. J. (1997). Phosphorylation of Na,K-ATPase by protein kinase C at Ser18 occurs in intact cells but does not result in direct inhibition of ATP hydrolysis. J. Biol. Chem. 272, 17726–17733. 10.1074/jbc.272.28.17726 9211924

[B89] Florea S. Anjak A. Cai W. F. Qian J. Vafiadaki E. Figueria S. (2012). Constitutive phosphorylation of inhibitor-1 at Ser67 and Thr75 depresses calcium cycling in cardiomyocytes and leads to remodeling upon aging. Basic Res. Cardiol. 107, 279. 10.1007/s00395-012-0279-z 22777184 PMC3587163

[B90] Force T. Kuida K. Namchuk M. Parang K. Kyriakis J. M. (2004). Inhibitors of protein kinase signaling pathways: emerging therapies for cardiovascular disease. Circulation 109, 1196–1205. 10.1161/01.CIR.0000118538.21306.A9 15023894

[B91] Gerhardstein B. L. Puri T. S. Chien A. J. Hosey M. M. (1999). Identification of the sites phosphorylated by cyclic AMP-dependent protein kinase on the β 2 subunit of L-type voltage-dependent calcium channels. Biochemistry 38, 10361–10370. 10.1021/bi990896o 10441130

[B92] Gomez del Arco P. Martinez-Martinez S. Maldonado J. L. Ortega-Perez I. Redondo J. M. (2000). A role for the p38 MAP kinase pathway in the nuclear shuttling of NFATp. J. Biol. Chem. 275, 13872–13878. 10.1074/jbc.275.18.13872 10788511

[B93] Gray C. B. Heller Brown J. (2014). CaMKIIdelta subtypes: localization and function. Front. Pharmacol. 5:15. 10.3389/fphar.2014.00015 24575042 PMC3920101

[B94] Greiser M. Neuberger H. R. Harks E. El-Armouche A. Boknik P. De Haan S. (2009). Distinct contractile and molecular differences between two goat models of atrial dysfunction: AV block-induced atrial dilatation and atrial fibrillation. J. Mol. Cell Cardiol. 46, 385–394. 10.1016/j.yjmcc.2008.11.012 19100271

[B95] Grueter C. E. Abiria S. A. Dzhura I. Wu Y. Ham A. J. Mohler P. J. (2006). L-type Ca^2+^ channel facilitation mediated by phosphorylation of the β subunit by CaMKII. Mol. Cell. 23, 641–650. 10.1016/j.molcel.2006.07.006 16949361

[B96] Gu J. Noe A. Chandra P. Al-Fayoumi S. Ligueros-Saylan M. Sarangapani R. (2010). Pharmacokinetics and pharmacodynamics of LCZ696, a novel dual-acting angiotensin receptor-neprilysin inhibitor (ARNi). J. Clin. Pharmacol. 50, 401–414. 10.1177/0091270009343932 19934029

[B97] Gu Q. Jin N. Sheng H. Yin X. Zhu J. (2011). Cyclic AMP-dependent protein kinase A regulates the alternative splicing of CaMKIIdelta. PLoS ONE 6:e25745. 10.1371/journal.pone.0025745 22132070 PMC3222655

[B98] Gupta R. C. Mishra S. Rastogi S. Imai M. Habib O. Sabbah H. N. (2003). Cardiac SR-coupled PP1 activity and expression are increased and inhibitor 1 protein expression is decreased in failing hearts. Am. J. Physiol. Heart Circ. Physiol. 285, H2373–H2381. 10.1152/ajpheart.00442.2003 14613911

[B99] Gwack Y. Sharma S. Nardone J. Tanasa B. Iuga A. Srikanth S. (2006). A genome-wide Drosophila RNAi screen identifies DYRK-family kinases as regulators of NFAT. Nature 441, 646–650. 10.1038/nature04631 16511445

[B100] Hall D. D. Feekes J. A. Arachchige Don A. S. Shi M. Hamid J. Chen L. (2006). Binding of protein phosphatase 2A to the L-type calcium channel Cav1.2 next to Ser1928, its main PKA site, is critical for Ser1928 dephosphorylation. Biochemistry 45, 3448–3459. 10.1021/bi051593z 16519540

[B101] Harvey R. D. (2004). Regulation of cardiac Na-Ca exchange activity by selective tyrosine kinase inhibition. Br. J. Pharmacol. 143, 929–930. 10.1038/sj.bjp.0706009 15545292 PMC1575961

[B102] Heijman J. Dewenter M. El-Armouche A. Dobrev D. (2013). Function and regulation of serine/threonine phosphatases in the healthy and diseased heart. J. Mol. Cell Cardiol. 64, 90–98. 10.1016/j.yjmcc.2013.09.006 24051368

[B103] Heineke J. Molkentin J. D. (2006). Regulation of cardiac hypertrophy by intracellular signalling pathways. Nat. Rev. Mol. Cell Biol. 7, 589–600. 10.1038/nrm1983 16936699

[B104] Heineke J. Ritter O. (2012). Cardiomyocyte calcineurin signaling in subcellular domains: from the sarcolemma to the nucleus and beyond. J. Mol. Cell Cardiol. 52, 62–73. 10.1016/j.yjmcc.2011.10.018 22064325

[B105] Hemmings H. C. Jr. Girault J. A. Nairn A. C. Bertuzzi G. Greengard P. (1992). Distribution of protein phosphatase inhibitor-1 in brain and peripheral tissues of various species: comparison with DARPP-32. J. Neurochem. 59, 1053–1061. 10.1111/j.1471-4159.1992.tb08347.x 1353788

[B106] Herzig S. Neumann J. (2000). Effects of serine/threonine protein phosphatases on ion channels in excitable membranes. Physiol. Rev. 80, 173–210. 10617768 10.1152/physrev.2000.80.1.173

[B107] Hescheler J. Mieskes G. Ruegg J. C. Takai A. Trautwein W. (1988). Effects of a protein phosphatase inhibitor, okadaic acid, on membrane currents of isolated guinea-pig cardiac myocytes. Pflugers. Arch. 412, 248–252. 10.1007/BF00582504 2847114

[B108] Hong T. T. Smyth J. W. Chu K. Y. Vogan J. M. Fong T. S. Jensen B. C. (2012). BIN1 is reduced and Cav1.2 trafficking is impaired in human failing cardiomyocytes. Heart Rhythm 9, 812–820. 10.1016/j.hrthm.2011.11.055 22138472 PMC3306544

[B109] Hsieh-Wilson L. C. Allen P. B. Watanabe T. Nairn A. C. Greengard P. (1999). Characterization of the neuronal targeting protein spinophilin and its interactions with protein phosphatase-1. Biochemistry 38, 4365–4373. 10.1021/bi982900m 10194355

[B110] Huang B. Wang S. Qin D. Boutjdir M. El-Sherif N. (1999). Diminished basal phosphorylation level of phospholamban in the postinfarction remodeled rat ventricle: role of β-adrenergic pathway, G(i) protein, phosphodiesterase, and phosphatases. Circ. Res. 85, 848–855. 10.1161/01.RES.85.9.848 10532953

[B111] Huang C. Cao W. Liao R. Wang J. Wang Y. Tong L. (2014). PP1γ functionally augments the alternative splicing of CaMKIIdelta through interaction with ASF. Am. J. Physiol. Cell Physiol. 306, C167–C177. 10.1152/ajpcell.00145.2013 24196533

[B112] Huang F. L. Glinsmann W. H. (1976). Separation and characterization of two phosphorylase phosphatase inhibitors from rabbit skeletal muscle. Eur. J. Biochem. 70, 419–426. 10.1111/j.1432-1033.1976.tb11032.x 188646

[B113] Huang K. X. Paudel H. K. (2000). Ser67-phosphorylated inhibitor 1 is a potent protein phosphatase 1 inhibitor. Proc. Natl. Acad. Sci. U.S.A. 97, 5824–5829. 10.1073/pnas.100460897 10811908 PMC18518

[B114] Huang Y. C. Chen Y. C. Tsay H. J. Chyan C. L. Chen C. Y. Huang H. B. (2010). The effect of PKA-phosphorylation on the structure of inhibitor-1 studied by NMR spectroscopy. J. Biochem. 147, 273–278. 10.1093/jb/mvp178 19887526

[B115] Hu C. Depuy S. D. Yao J. Mcintire W. E. Barrett P. Q. (2009). Protein kinase A activity controls the regulation of T-type CaV3.2 channels by Gβγ dimers. J. Biol. Chem. 284, 7465–7473. 10.1074/jbc.M808049200 19131331 PMC2658042

[B116] Hulme J. T. Westenbroek R. E. Scheuer T. Catterall W. A. (2006). Phosphorylation of serine 1928 in the distal C-terminal domain of cardiac CaV1.2 channels during β1-adrenergic regulation. Proc. Natl. Acad. Sci. U.S.A. 103, 16574–16579. 10.1073/pnas.0607294103 17053072 PMC1637623

[B117] Humphries K. M. Deal M. S. Taylor S. S. (2005). Enhanced dephosphorylation of cAMP-dependent protein kinase by oxidation and thiol modification. J. Biol. Chem. 280, 2750–2758. 10.1074/jbc.M410242200 15533936

[B118] Hund T. J. Koval O. M. Li J. Wright P. J. Qian L. Snyder J. S. (2010). A β(IV)-spectrin/CaMKII signaling complex is essential for membrane excitability in mice. J. Clin. Invest. 120, 3508–3519. 10.1172/JCI43621 20877009 PMC2947241

[B119] Iacobazzi D. Garaeva I. Albertario A. Cherif M. Angelini G. D. Caputo M. (2015). Protein phosphatase 1 β is modulated by chronic hypoxia and involved in the angiogenic endothelial cell migration. Cell Physiol. Biochem 36, 384–394. 10.1159/000430257 25967976

[B120] Ishikawa K. Fish K. M. Tilemann L. Rapti K. Aguero J. Santos-Gallego C. G. (2014). Cardiac I-1c overexpression with reengineered AAV improves cardiac function in swine ischemic heart failure. Mol. Ther. 22, 2038–2045. 10.1038/mt.2014.127 25023328 PMC4429688

[B121] Jackson W. A. Colyer J. (1996). Translation of Ser16 and Thr17 phosphorylation of phospholamban into Ca^2+^-pump stimulation. Biochem. J. 316, 201–207. 10.1042/bj3160201 8645206 PMC1217323

[B122] Jalife J. Anumonwo J. M. Berenfeld O. (2003). Toward an understanding of the molecular mechanisms of ventricular fibrillation. J. Interv. Card. Electrophysiol. 9, 119–129. 10.1023/A:1026215919730 14574022

[B123] Janssens V. Goris J. (2001). Protein phosphatase 2A: a highly regulated family of serine/threonine phosphatases implicated in cell growth and signalling. Biochem. J. 353, 417–439. 10.1042/bj3530417 11171037 PMC1221586

[B124] Jeong A. L. Yang Y. (2013). PP2A function toward mitotic kinases and substrates during the cell cycle. BMB Rep. 46, 289–294. 10.5483/BMBRep.2013.46.6.041 23790971 PMC4133904

[B125] Jespersen T. Gavillet B. Van Bemmelen M. X. Cordonier S. Thomas M. A. Staub O. (2006). Cardiac sodium channel Na(v)1.5 interacts with and is regulated by the protein tyrosine phosphatase PTPH1. Biochem. Biophys. Res. Commun. 348, 1455–1462. 10.1016/j.bbrc.2006.08.014 16930557

[B126] Jessup M. Fox K. A. Komajda M. Mcmurray J. J. Packer M. (2014). PARADIGM-HF–the experts’ discussion. N. Engl. J. Med. 371, e15. 10.1056/NEJMp1410203 25184757

[B127] Jeyaraman M. Tanguy S. Fandrich R. R. Lukas A. Kardami E. (2003). Ischemia-induced dephosphorylation of cardiomyocyte connexin-43 is reduced by okadaic acid and calyculin A but not fostriecin. Mol. Cell. Biochem. 242, 129–134. 10.1023/A:1021102131603 12619875

[B128] Jideama N. M. Crawford B. H. Hussain A. K. Raynor R. L. (2006). Dephosphorylation specificities of protein phosphatase for cardiac troponin I, troponin T, and sites within troponin T. Int. J. Biol. Sci. 2, 1–9. 10.7150/ijbs.2.1 16585947 PMC1415850

[B129] Johnson L. N. (2009). The regulation of protein phosphorylation. Biochem. Soc. Trans. 37, 627–641. 10.1042/BST0370627 19614568

[B130] Kamp T. J. Hell J. W. (2000). Regulation of cardiac L-type calcium channels by protein kinase A and protein kinase C. Circ. Res. 87, 1095–1102. 10.1161/01.RES.87.12.1095 11110765

[B131] Keen J. C. Garrett-Mayer E. Pettit C. Mack K. M. Manning J. Herman J. G. (2004). Epigenetic regulation of protein phosphatase 2A (PP2A), lymphotactin (XCL1) and estrogen receptor (ER) expression in human breast cancer cells. Cancer Biol. Ther. 3, 1304–1312. 10.4161/cbt.3.12.1458 15662126

[B132] Kimura T. Han W. Pagel P. Nairn A. C. Caplan M. J. (2011). Protein phosphatase 2A interacts with the Na,K-ATPase and modulates its trafficking by inhibition of its association with arrestin. PLoS ONE 6:e29269. 10.1371/journal.pone.0029269 22242112 PMC3248462

[B133] Kirchner J. Gross S. Bennett D. Alphey L. (2007). Essential, overlapping and redundant roles of the Drosophila protein phosphatase 1 and 1 β genes. Genetics 176, 273–281. 10.1534/genetics.106.069914 17513890 PMC1893066

[B134] Kolwicz S. C. Jr. Purohit S. Tian R. (2013). Cardiac metabolism and its interactions with contraction, growth, and survival of cardiomyocytes. Circ. Res. 113, 603–616. 10.1161/CIRCRESAHA.113.302095 23948585 PMC3845521

[B135] Korrodi-Gregorio L. Esteves S. L. Fardilha M. (2014). Protein phosphatase 1 catalytic isoforms: specificity toward interacting proteins. Transl. Res. 164, 366–391. 10.1016/j.trsl.2014.07.001 25090308

[B136] Kreusser M. M. Backs J. (2014). Integrated mechanisms of CaMKII-dependent ventricular remodeling. Front. Pharmacol. 5:36. 10.3389/fphar.2014.00036 24659967 PMC3950490

[B137] Kwon Y. G. Huang H. B. Desdouits F. Girault J. A. Greengard P. Nairn A. C. (1997). Characterization of the interaction between DARPP-32 and protein phosphatase 1 (PP-1): DARPP-32 peptides antagonize the interaction of PP-1 with binding proteins. Proc. Natl. Acad. Sci. U.S.A. 94, 3536–3541. 10.1073/pnas.94.8.3536 9108011 PMC20474

[B138] Lauriol J. Jaffre F. Kontaridis M. I. (2015). The role of the protein tyrosine phosphatase SHP2 in cardiac development and disease. Semin. Cell Dev. Biol. 37, 73–81. 10.1016/j.semcdb.2014.09.013 25256404 PMC4339543

[B139] Lee T. S. Karl R. Moosmang S. Lenhardt P. Klugbauer N. Hofmann F. (2006). Calmodulin kinase II is involved in voltage-dependent facilitation of the L-type Cav1.2 calcium channel: identification of the phosphorylation sites. J. Biol. Chem. 281, 25560–25567. 10.1074/jbc.M508661200 16820363

[B140] Lei M. Wang X. Ke Y. Solaro R. J. (2015). Regulation of Ca^2+^ transient by PP2A in normal and failing heart. Front Physiol 6:13. 10.3389/fphys.2015.00013 25688213 PMC4310266

[B141] Lemke T. Welling A. Christel C. J. Blaich A. Bernhard D. Lenhardt P. (2008). Unchanged β-adrenergic stimulation of cardiac L-type calcium channels in Ca v 1.2 phosphorylation site S1928A mutant mice. J. Biol. Chem. 283, 34738–34744. 10.1074/jbc.M804981200 18829456 PMC3259877

[B142] Li B. Kaetzel M. A. Dedman J. R. (2006). Signaling pathways regulating murine cardiac CREB phosphorylation. Biochem. Biophys. Res. Commun. 350, 179–184. 10.1016/j.bbrc.2006.09.016 16996475

[B143] Li X. Wilmanns M. Thornton J. Kohn M. (2013). Elucidating human phosphatase-substrate networks. Sci. Signal. 6, rs10. 10.1126/scisignal.2003203 23674824

[B144] Lim H. W. Molkentin J. D. (1999). Calcineurin and human heart failure. Nat. Med. 5, 246–247. 10.1038/6430 10086361

[B145] Ling S. Sun Q. Li Y. Zhang L. Zhang P. Wang X. (2012). CKIP-1 inhibits cardiac hypertrophy by regulating class II histone deacetylase phosphorylation through recruiting PP2A. Circulation 126, 3028–3040. 10.1161/CIRCULATIONAHA.112.102780 23151343

[B146] Liu B. Ho H. T. Velez-Cortes F. Lou Q. Valdivia C. R. Knollmann B. C. (2014). Genetic ablation of ryanodine receptor 2 phosphorylation at Ser-2808 aggravates Ca^2+^-dependent cardiomyopathy by exacerbating diastolic Ca^2+^ release. J. Physiol. 592, 1957–1973. 10.1113/jphysiol.2013.264689 24445321 PMC4230772

[B147] Liu C. L. Li X. Hu G. L. Li R. J. He Y. Y. Zhong W. (2012). Salubrinal protects against tunicamycin and hypoxia induced cardiomyocyte apoptosis via the PERK-eIF2 signaling pathway. J Geriatr. Cardiol. 9, 258–268. 10.3724/SP.J.1263.2012.02292 23097656 PMC3470025

[B148] Liu C. W. Wang R. H. Dohadwala M. Schonthal A. H. Villa-Moruzzi E. Berndt N. (1999). Inhibitory phosphorylation of PP1 catalytic subunit during the G(1)/S transition. J. Biol. Chem. 274, 29470–29475. 10.1074/jbc.274.41.29470 10506210

[B149] Liu J. Sirenko S. Juhaszova M. Ziman B. Shetty V. Rain S. (2011). A full range of mouse sinoatrial node AP firing rates requires protein kinase A-dependent calcium signaling. J. Mol. Cell Cardiol. 51, 730–739. 10.1016/j.yjmcc.2011.07.028 21840316 PMC3184386

[B149a] Liu R. Correll R. N. Davis J. Vagnozzi R. J. York A. J. Sargent M. A. (2015). Cardiac-specific deletion of protein phosphatase 1β promotes increased myofilament protein phosphorylation and contractile alterations. J. Mol. Cell. Cardiol. 87, 204–213. 10.1016/j.yjmcc.2015.08.018 [Epub ahead of print]. 26334248 PMC4637224

[B150] Liu Y. Schneider M. F. (2013). Opposing HDAC4 nuclear fluxes due to phosphorylation by β-adrenergic activated protein kinase A or by activity or Epac activated CaMKII in skeletal muscle fibres. J. Physiol. 591, 3605–3623. 10.1113/jphysiol.2013.256263 23652597 PMC3731617

[B151] Luo M. Anderson M. E. (2013). Mechanisms of altered Ca^2+^ handling in heart failure. Circ. Res. 113, 690–708. 10.1161/CIRCRESAHA.113.301651 23989713 PMC4080816

[B152] Luo W. Grupp I. L. Harrer J. Ponniah S. Grupp G. Duffy J. J. (1994). Targeted ablation of the phospholamban gene is associated with markedly enhanced myocardial contractility and loss of β-agonist stimulation. Circ. Res. 75, 401–409. 10.1161/01.RES.75.3.401 8062415

[B153] Luss H. Klein-Wiele O. Boknik P. Herzig S. Knapp J. Linck B. (2000). Regional expression of protein phosphatase type 1 and 2A catalytic subunit isoforms in the human heart. J. Mol. Cell Cardiol. 32, 2349–2359. 10.1006/jmcc.2000.1265 11113010

[B154] MacDougall L. K. Jones L. R. Cohen P. (1991). Identification of the major protein phosphatases in mammalian cardiac muscle which dephosphorylate phospholamban. Eur. J. Biochem. 196, 725–734. 10.1111/j.1432-1033.1991.tb15871.x 1849481

[B155] Maillet M. Purcell N. H. Sargent M. A. York A. J. Bueno O. F. Molkentin J. D. (2008). DUSP6 (MKP3) null mice show enhanced ERK1/2 phosphorylation at baseline and increased myocyte proliferation in the heart affecting disease susceptibility. J. Biol. Chem. 283, 31246–31255. 10.1074/jbc.M806085200 18753132 PMC2576531

[B156] Marionneau C. Lichti C. F. Lindenbaum P. Charpentier F. Nerbonne J. M. Townsend R. R. (2012). Mass spectrometry-based identification of native cardiac Nav1.5 channel subunit phosphorylation sites. J. Proteome Res. 11, 5994–6007. 10.1021/pr300702c 23092124 PMC3518584

[B157] Marks A. R. Marx S. O. Reiken S. (2002). Regulation of ryanodine receptors via macromolecular complexes: a novel role for leucine/isoleucine zippers. Trends Cardiovasc. Med. 12, 166–170. 10.1016/S1050-1738(02)00156-1 12069756

[B158] Marx S. O. Kurokawa J. Reiken S. Motoike H. D’armiento J. Marks A. R. (2002). Requirement of a macromolecular signaling complex for β adrenergic receptor modulation of the KCNQ1-KCNE1 potassium channel. Science 295, 496–499. 10.1126/science.1066843 11799244

[B159] Marx S. O. Reiken S. Hisamatsu Y. Gaburjakova M. Gaburjakova J. Yang Y. M. (2001). Phosphorylation-dependent regulation of ryanodine receptors: a novel role for leucine/isoleucine zippers. J. Cell Biol. 153, 699–708. 10.1083/jcb.153.4.699 11352932 PMC2192391

[B160] Marx S. O. Reiken S. Hisamatsu Y. Jayaraman T. Burkhoff D. Rosemblit N. (2000). PKA phosphorylation dissociates FKBP12.6 from the calcium release channel (ryanodine receptor): defective regulation in failing hearts. Cell 101, 365–376. 10.1016/S0092-8674(00)80847-8 10830164

[B161] McAvoy T. Allen P. B. Obaishi H. Nakanishi H. Takai Y. Greengard P. (1999). Regulation of neurabin I interaction with protein phosphatase 1 by phosphorylation. Biochemistry 38, 12943–12949. 10.1021/bi991227d 10504266

[B162] McConnell J. L. Wadzinski B. E. (2009). Targeting protein serine/threonine phosphatases for drug development. Mol. Pharmacol. 75, 1249–1261. 10.1124/mol.108.053140 19299564 PMC2684880

[B163] Meng X. Xiao B. Cai S. Huang X. Li F. Bolstad J. (2007). Three-dimensional localization of serine 2808, a phosphorylation site in cardiac ryanodine receptor. J. Biol. Chem. 282, 25929–25939. 10.1074/jbc.M704474200 17606610 PMC2796423

[B164] Miller C. Zhang M. He Y. Zhao J. Pelletier J. P. Martel-Pelletier J. (1998). Transcriptional induction of cyclooxygenase-2 gene by okadaic acid inhibition of phosphatase activity in human chondrocytes: co-stimulation of AP-1 and CRE nuclear binding proteins. J. Cell. Biochem. 69, 392–413. 9620167

[B165] Minobe E. Maeda S. Xu J. Hao L. Kameyama A. Kameyama M. (2014). A new phosphorylation site in cardiac L-type Ca^2+^ channels (Cav1.2) responsible for its cAMP-mediated modulation. Am. J. Physiol. Cell Physiol. 307, C999–C1009. 10.1152/ajpcell.00267.2014 25209265

[B166] Mirtschink P. Krishnan J. Grimm F. Sarre A. Horl M. Kayikci M. (2015). HIF-driven SF3B1 induces KHK-C to enforce fructolysis and heart disease. Nature 522, 444–449. 10.1038/nature14508 26083752 PMC4783869

[B167] Mohamed A. S. Dignam J. D. Schlender K. K. (1998). Cardiac myosin-binding protein C (MyBP-C): identification of protein kinase A and protein kinase C phosphorylation sites. Arch. Biochem. Biophys. 358, 313–319. 10.1006/abbi.1998.0857 9784245

[B168] Molkentin J. D. (2000). Calcineurin and beyond: cardiac hypertrophic signaling. Circ. Res. 87, 731–738. 10.1161/01.RES.87.9.731 11055975

[B169] Molkentin J. D. Lu J. R. Antos C. L. Markham B. Richardson J. Robbins J. (1998). A calcineurin-dependent transcriptional pathway for cardiac hypertrophy. Cell 93, 215–228. 10.1016/S0092-8674(00)81573-1 9568714 PMC4459646

[B170] Montminy M. R. Gonzalez G. A. Yamamoto K. K. (1990). Characteristics of the cAMP response unit. Metabolism 39, 6–12. 10.1016/0026-0495(90)90198-L 2169573

[B171] Moorhead G. B. Trinkle-Mulcahy L. Ulke-Lemee A. (2007). Emerging roles of nuclear protein phosphatases. Nat. Rev. Mol. Cell Biol. 8, 234–244. 10.1038/nrm2126 17318227

[B172] Murnion M. E. Adams R. R. Callister D. M. Allis C. D. Earnshaw W. C. Swedlow J. R. (2001). Chromatin-associated protein phosphatase 1 regulates aurora-B and histone H3 phosphorylation. J. Biol. Chem. 276, 26656–26665. 10.1074/jbc.M102288200 11350965

[B173] Murray K. T. Hu N. N. Daw J. R. Shin H. G. Watson M. T. Mashburn A. B. (1997). Functional effects of protein kinase C activation on the human cardiac Na^+^ channel. Circ. Res. 80, 370–376. 10.1161/01.RES.80.3.370 9048657

[B174] Narayanan N. Xu A. (1997). Phosphorylation and regulation of the Ca^2+^-pumping ATPase in cardiac sarcoplasmic reticulum by calcium/calmodulin-dependent protein kinase. Basic Res. Cardiol. 92(Suppl. 1), 25–35. 10.1007/BF00794065 9202841

[B175] Nattel S. Burstein B. Dobrev D. (2008). Atrial remodeling and atrial fibrillation: mechanisms and implications. Circ. Arrhythm. Electrophysiol. 1, 62–73. 10.1161/CIRCEP.107.754564 19808395

[B176] Neef S. Dybkova N. Sossalla S. Ort K. R. Fluschnik N. Neumann K. (2010). CaMKII-dependent diastolic SR Ca^2+^ leak and elevated diastolic Ca^2+^ levels in right atrial myocardium of patients with atrial fibrillation. Circ. Res. 106, 1134–1144. 10.1161/CIRCRESAHA.109.203836 20056922

[B177] Neuber C. Uebeler J. Schulze T. Sotoud H. El-Armouche A. Eschenhagen T. (2014). Guanabenz interferes with ER stress and exerts protective effects in cardiac myocytes. PLoS ONE 9:e98893. 10.1371/journal.pone.0098893 24892553 PMC4044035

[B178] Neumann J. Eschenhagen T. Jones L. R. Linck B. Schmitz W. Scholz H. (1997). Increased expression of cardiac phosphatases in patients with end-stage heart failure. J. Mol. Cell Cardiol. 29, 265–272. 10.1006/jmcc.1996.0271 9040041

[B179] Neumann J. Maas R. Boknik P. Jones L. R. Zimmermann N. Scholz H. (1999). Pharmacological characterization of protein phosphatase activities in preparations from failing human hearts. J. Pharmacol. Exp. Ther. 289, 188–193. 10087003

[B180] Nicolaou P. Hajjar R. J. Kranias E. G. (2009). Role of protein phosphatase-1 inhibitor-1 in cardiac physiology and pathophysiology. J. Mol. Cell Cardiol. 47, 365–371. 10.1016/j.yjmcc.2009.05.010 19481088 PMC2716438

[B181] Nicolas C. S. Park K. H. El Harchi A. Camonis J. Kass R. S. Escande D. (2008). IKs response to protein kinase A-dependent KCNQ1 phosphorylation requires direct interaction with microtubules. Cardiovasc. Res. 79, 427–435. 10.1093/cvr/cvn085 18390900 PMC2781743

[B182] Nikandrova Y. A. Jiao Y. Baucum A. J. Tavalin S. J. Colbran R. J. (2010). Ca^2+^/calmodulin-dependent protein kinase II binds to and phosphorylates a specific SAP97 splice variant to disrupt association with AKAP79/150 and modulate -amino-3-hydroxy-5-methyl-4-isoxazolepropionic acid-type glutamate receptor (AMPAR) activity. J. Biol. Chem. 285, 923–934. 10.1074/jbc.M109.033985 19858198 PMC2801293

[B183] Novak B. Kapuy O. Domingo-Sananes M. R. Tyson J. J. (2010). Regulated protein kinases and phosphatases in cell cycle decisions. Curr. Opin. Cell Biol. 22, 801–808. 10.1016/j.ceb.2010.07.001 20678910 PMC3769698

[B184] Okamura H. Garcia-Rodriguez C. Martinson H. Qin J. Virshup D. M. Rao A. (2004). A conserved docking motif for CK1 binding controls the nuclear localization of NFAT1. Mol. Cell. Biol. 24, 4184–4195. 10.1128/MCB.24.10.4184-4195.2004 15121840 PMC400483

[B185] Oka T. Dai Y. S. Molkentin J. D. (2005). Regulation of calcineurin through transcriptional induction of the calcineurin A β promoter in vitro and in vivo. Mol. Cell. Biol. 25, 6649–6659. 10.1128/MCB.25.15.6649-6659.2005 16024800 PMC1190362

[B186] Oliver C. J. Terry-Lorenzo R. T. Elliott E. Bloomer W. A. Li S. Brautigan D. L. (2002). Targeting protein phosphatase 1 (PP1) to the actin cytoskeleton: the neurabin I/PP1 complex regulates cell morphology. Mol. Cell. Biol. 22, 4690–4701. 10.1128/MCB.22.13.4690-4701.2002 12052877 PMC133892

[B187] Olsen J. V. Blagoev B. Gnad F. Macek B. Kumar C. Mortensen P. (2006). Global, in vivo, and site-specific phosphorylation dynamics in signaling networks. Cell 127, 635–648. 10.1016/j.cell.2006.09.026 17081983

[B188] Otmakhov N. Regmi S. Lisman J. E. (2015). Fast decay of CaMKII FRET sensor signal in spines after LTP induction is not due to its dephosphorylation. PLoS ONE 10:e0130457. 10.1371/journal.pone.0130457 26086939 PMC4472229

[B189] Packer M. (1985). Sudden unexpected death in patients with congestive heart failure: a second frontier. Circulation 72, 681–685. 10.1161/01.CIR.72.4.681 2863012

[B190] Palmer C. J. Scott B. T. Jones L. R. (1991). Purification and complete sequence determination of the major plasma membrane substrate for cAMP-dependent protein kinase and protein kinase C in myocardium. J. Biol. Chem. 266, 11126–11130. 1710217

[B191] Palmeri A. Ferre F. Helmer-Citterich M. (2014). Exploiting holistic approaches to model specificity in protein phosphorylation. Front. Genet. 5:315. 10.3389/fgene.2014.00315 25324856 PMC4179730

[B192] Pan M. G. Xiong Y. Chen F. (2013). NFAT gene family in inflammation and cancer. Curr. Mol. Med. 13, 543–554. 10.2174/1566524011313040007 22950383 PMC3694398

[B193] Pare G. C. Bauman A. L. Mchenry M. Michel J. J. Dodge-Kafka K. L. Kapiloff M. S. (2005). The mAKAP complex participates in the induction of cardiac myocyte hypertrophy by adrenergic receptor signaling. J. Cell Sci. 118, 5637–5646. 10.1242/jcs.02675 16306226

[B194] Paroni G. Cernotta N. Dello Russo C. Gallinari P. Pallaoro M. Foti C. (2008). PP2A regulates HDAC4 nuclear import. Mol. Biol. Cell 19, 655–667. 10.1091/mbc.E07-06-0623 18045992 PMC2230598

[B195] Pathak A. Del Monte F. Zhao W. Schultz J. E. Lorenz J. N. Bodi I. (2005). Enhancement of cardiac function and suppression of heart failure progression by inhibition of protein phosphatase 1. Circ. Res. 96, 756–766. 10.1161/01.RES.0000161256.85833.fa 15746443

[B196] Patterson K. I. Brummer T. O’brien P. M. Daly R. J. (2009). Dual-specificity phosphatases: critical regulators with diverse cellular targets. Biochem. J. 418, 475–489. 10.1042/BJ20082234 19228121

[B197] Peti W. Nairn A. C. Page R. (2013). Structural basis for protein phosphatase 1 regulation and specificity. FEBS J. 280, 596–611. 10.1111/j.1742-4658.2012.08509.x 22284538 PMC3350600

[B198] Petzhold D. Da Costa-Goncalves A. C. Gross V. Morano I. (2011). Spinophilin is required for normal morphology, Ca^2+^ homeostasis and contraction but dispensable for β-adrenergic stimulation of adult cardiomyocytes. J. Muscle Res. Cell Motil. 32, 243–248. 10.1007/s10974-011-9259-4 21922228

[B199] Prystowsky E. N. Padanilam B. J. Joshi S. Fogel R. I. (2012). Ventricular arrhythmias in the absence of structural heart disease. J. Am. Coll. Cardiol. 59, 1733–1744. 10.1016/j.jacc.2012.01.036 22575310

[B200] Pulido R. Hooft van Huijsduijnen R. (2008). Protein tyrosine phosphatases: dual-specificity phosphatases in health and disease. FEBS J. 275, 848–866. 10.1111/j.1742-4658.2008.06250.x 18298792

[B201] Qu Z. Weiss J. N. (2015). Mechanisms of ventricular arrhythmias: from molecular fluctuations to electrical turbulence. Annu. Rev. Physiol. 77, 29–55. 10.1146/annurev-physiol-021014-071622 25340965 PMC4342983

[B202] Raghavan S. Williams I. Aslam H. Thomas D. Szoor B. Morgan G. (2000). Protein phosphatase 1β is required for the maintenance of muscle attachments. Curr. Biol. 10, 269–272. 10.1016/S0960-9822(00)00364-X 10712908

[B203] Ragusa M. J. Allaire M. Nairn A. C. Page R. Peti W. (2011). Flexibility in the PP1:spinophilin holoenzyme. FEBS Lett. 585, 36–40. 10.1016/j.febslet.2010.11.022 21094159 PMC3017638

[B204] Ragusa M. J. Dancheck B. Critton D. A. Nairn A. C. Page R. Peti W. (2010). Spinophilin directs protein phosphatase 1 specificity by blocking substrate binding sites. Nat. Struct. Mol. Biol. 17, 459–464. 10.1038/nsmb.1786 20305656 PMC2924587

[B205] Rapundalo S. T. (1998). Cardiac protein phosphorylation: functional and pathophysiological correlates. Cardiovasc. Res. 38, 559–588. 10.1016/S0008-6363(98)00063-7 9747427

[B206] Redden J. M. Dodge-Kafka K. L. (2011). AKAP phosphatase complexes in the heart. J. Cardiovasc. Pharmacol. 58, 354–362. 10.1097/FJC.0b013e31821e5649 21562429 PMC3158305

[B207] Reese L. C. Laezza F. Woltjer R. Taglialatela G. (2011). Dysregulated phosphorylation of Ca^2+^ /calmodulin-dependent protein kinase II- in the hippocampus of subjects with mild cognitive impairment and Alzheimer’s disease. J. Neurochem. 119, 791–804. 10.1111/j.1471-4159.2011.07447.x 21883216 PMC4021864

[B208] Reiken S. Gaburjakova M. Guatimosim S. Gomez A. M. D’armiento J. Burkhoff D. (2003). Protein kinase A phosphorylation of the cardiac calcium release channel (ryanodine receptor) in normal and failing hearts. Role of phosphatases and response to isoproterenol. J. Biol. Chem. 278, 444–453. 10.1074/jbc.M207028200 12401811

[B209] Reither G. Chatterjee J. Beullens M. Bollen M. Schultz C. Kohn M. (2013). Chemical activators of protein phosphatase-1 induce calcium release inside intact cells. Chem. Biol. 20, 1179–1186. 10.1016/j.chembiol.2013.07.008 23972940

[B210] Remo B. F. Giovannone S. Fishman G. I. (2012). Connexin43 cardiac gap junction remodeling: lessons from genetically engineered murine models. J. Membr. Biol. 245, 275–281. 10.1007/s00232-012-9448-0 22722763 PMC3630470

[B211] Roberts-Thomson K. C. Lau D. H. Sanders P. (2011). The diagnosis and management of ventricular arrhythmias. Nat. Rev. Cardiol. 8, 311–321. 10.1038/nrcardio.2011.15 21343901

[B212] Roskoski R. Jr. (2015). A historical overview of protein kinases and their targeted small molecule inhibitors. Pharmacol. Res. 100, 1–23. 10.1016/j.phrs.2015.07.010 26207888

[B213] Rossow C. F. Dilly K. W. Santana L. F. (2006). Differential calcineurin/NFATc3 activity contributes to the ito transmural gradient in the mouse heart. Circ. Res. 98, 1306–1313. 10.1161/01.RES.0000222028.92993.10 16614306

[B214] Rossow C. F. Minami E. Chase E. G. Murry C. E. Santana L. F. (2004). NFATc3-induced reductions in voltage-gated K^+^ currents after myocardial infarction. Circ. Res. 94, 1340–1350. 10.1161/01.RES.0000128406.08418.34 15087419

[B215] Roy J. Cyert M. S. (2009). Cracking the phosphatase code: docking interactions determine substrate specificity. Sci. Signal. 2, re9. 10.1126/scisignal.2100re9 19996458

[B216] Sakisaka T. Nakanishi H. Takahashi K. Mandai K. Miyahara M. Satoh A. (1999). Different behavior of l-afadin and neurabin-II during the formation and destruction of cell–cell adherens junction. Oncogene 18, 1609–1617. 10.1038/sj.onc.1202451 10102631

[B217] Sato P. Y. Chuprun J. K. Schwartz M. Koch W. J. (2015). The evolving impact of g protein–coupled receptor kinases in cardiac health and disease. Physiol. Rev. 95, 377–404. 10.1152/physrev.00015.2014 25834229 PMC4551214

[B218] Sawicka A. Seiser C. (2014). Sensing core histone phosphorylation—a matter of perfect timing. Biochim. Biophys. Acta 1839, 711–718. 10.1016/j.bbagrm.2014.04.013 24747175 PMC4103482

[B219] Schillinger W. Fiolet J. W. Schlotthauer K. Hasenfuss G. (2003). Relevance of Na^+^-Ca^2+^ exchange in heart failure. Cardiovasc. Res. 57, 921–933. 10.1016/S0008-6363(02)00826-X 12650870

[B220] Schulze D. H. Muqhal M. Lederer W. J. Ruknudin A. M. (2003). Sodium/calcium exchanger (NCX1) macromolecular complex. J. Biol. Chem. 278, 28849–28855. 10.1074/jbc.M300754200 12754202

[B221] Schwinger R. H. Bundgaard H. Muller-Ehmsen J. Kjeldsen K. (2003). The Na, K-ATPase in the failing human heart. Cardiovasc. Res. 57, 913–920. 10.1016/S0008-6363(02)00767-8 12650869

[B222] Schwoerer A. P. Neuber C. Schmechel A. Melnychenko I. Mearini G. Boknik P. (2008). Mechanical unloading of the rat heart involves marked changes in the protein kinase-phosphatase balance. J. Mol. Cell Cardiol. 45, 846–852. 10.1016/j.yjmcc.2008.09.003 18848565

[B223] Sears D. Luong P. Yuan M. Nteliopoulos G. Man Y. K. Melo J. V. (2010). Functional phosphoproteomic analysis reveals cold-shock domain protein A to be a Bcr-Abl effector-regulating proliferation and transformation in chronic myeloid leukemia. Cell Death Dis. 1, e93. 10.1038/cddis.2010.72 21368869 PMC3032323

[B224] Sefton B. M. (2001). Overview of protein phosphorylation. Curr. Protoc. Cell Biol. 14. 10.1002/0471143030.cb1401s00 18228324

[B225] Senis Y. A. (2013). Protein-tyrosine phosphatases: a new frontier in platelet signal transduction. J. Thromb. Haemost. 11, 1800–1813. 10.1111/jth.12359 24015866

[B226] Sheridan C. M. Heist E. K. Beals C. R. Crabtree G. R. Gardner P. (2002). Protein kinase A negatively modulates the nuclear accumulation of NF-ATc1 by priming for subsequent phosphorylation by glycogen synthase kinase-3. J. Biol. Chem. 277, 48664–48676. 10.1074/jbc.M207029200 12351631

[B227] Shi J. Gu P. Zhu Z. Liu J. Chen Z. Sun X. (2012). Protein phosphatase 2A effectively modulates basal L-type Ca^2+^ current by dephosphorylating Ca(v)1.2 at serine 1866 in mouse cardiac myocytes. Biochem. Biophys. Res. Commun. 418, 792–798. 10.1016/j.bbrc.2012.01.105 22310722

[B228] Shigekawa M. Katanosaka Y. Wakabayashi S. (2007). Regulation of the cardiac Na^+^/Ca^2+^ exchanger by calcineurin and protein kinase C. Ann. N. Y. Acad. Sci. 1099, 53–63. 10.1196/annals.1387.059 17446445

[B229] Singh A. Redden J. M. Kapiloff M. S. Dodge-Kafka K. L. (2011). The large isoforms of A-kinase anchoring protein 18 mediate the phosphorylation of inhibitor-1 by protein kinase A and the inhibition of protein phosphatase 1 activity. Mol. Pharmacol. 79, 533–540. 10.1124/mol.110.065425 21149637 PMC3061358

[B230] Sipido K. R. Bito V. Antoons G. Volders P. G. Vos M. A. (2007). Na/Ca exchange and cardiac ventricular arrhythmias. Ann. N. Y. Acad. Sci. 1099, 339–348. 10.1196/annals.1387.066 17446474

[B231] Slupe A. M. Merrill R. A. Strack S. (2011). Determinants for substrate specificity of protein phosphatase 2A. Enzyme Res. 2011, 398751. 10.4061/2011/398751 21755039 PMC3132988

[B232] Solaro R. J. Kobayashi T. (2011). Protein phosphorylation and signal transduction in cardiac thin filaments. J. Biol. Chem. 286, 9935–9940. 10.1074/jbc.R110.197731 21257760 PMC3060547

[B233] Solomon S. D. Zile M. Pieske B. Voors A. Shah A. Kraigher-Krainer E. (2012). The angiotensin receptor neprilysin inhibitor LCZ696 in heart failure with preserved ejection fraction: a phase 2 double-blind randomised controlled trial. Lancet 380, 1387–1395. 10.1016/S0140-6736(12)61227-6 22932717

[B234] Sonnleitner A. Fleischer S. Schindler H. (1997). Gating of the skeletal calcium release channel by ATP is inhibited by protein phosphatase 1 but not by Mg^2+^. Cell Calcium 21, 283–290. 10.1016/S0143-4160(97)90116-0 9160164

[B235] Sotoud H. Borgmeyer U. Schulze C. El-Armouche A. Eschenhagen T. (2015). Development of phosphatase inhibitor-1 peptides acting as indirect activators of phosphatase 1. Naunyn Schmiedebergs Arch. Pharmacol. 388, 283–293. 10.1007/s00210-014-1065-2 25416155

[B236] Splawski I. Timothy K. W. Sharpe L. M. Decher N. Kumar P. Bloise R. (2004). Ca(V)1.2 calcium channel dysfunction causes a multisystem disorder including arrhythmia and autism. Cell 119, 19–31. 10.1016/j.cell.2004.09.011 15454078

[B237] Sumandea M. P. Steinberg S. F. (2011). Redox signaling and cardiac sarcomeres. J. Biol. Chem. 286, 9921–9927. 10.1074/jbc.R110.175489 21257753 PMC3060545

[B238] Sun H. Kerfant B. G. Zhao D. Trivieri M. G. Oudit G. Y. Penninger J. M. (2006). Insulin-like growth factor-1 and PTEN deletion enhance cardiac L-type Ca^2+^ currents via increased PI3K/PKB signaling. Circ. Res. 98, 1390–1397. 10.1161/01.RES.0000223321.34482.8c 16627784

[B239] Tajada S. Cidad P. Colinas O. Santana L. F. Lopez-Lopez J. R. Perez-Garcia M. T. (2013). Down-regulation of CaV1.2 channels during hypertension: how fewer CaV1.2 channels allow more Ca^2+^ into hypertensive arterial smooth muscle. J. Physiol. 591, 6175–6191. 10.1113/jphysiol.2013.265751 24167226 PMC3892470

[B240] Tandan S. Wang Y. Wang T. T. Jiang N. Hall D. D. Hell J. W. (2009). Physical and functional interaction between calcineurin and the cardiac L-type Ca^2+^ channel. Circ. Res. 105, 51–60. 10.1161/CIRCRESAHA.109.199828 19478199 PMC3038593

[B241] Tang Z. Z. Liao P. Li G. Jiang F. L. Yu D. Hong X. (2008). Differential splicing patterns of L-type calcium channel Cav1.2 subunit in hearts of Spontaneously Hypertensive Rats and Wistar Kyoto Rats. Biochim. Biophys. Acta 1783, 118–130. 10.1016/j.bbamcr.2007.11.003 18070605

[B242] Terrak M. Kerff F. Langsetmo K. Tao T. Dominguez R. (2004). Structural basis of protein phosphatase 1 regulation. Nature 429, 780–784. 10.1038/nature02582 15164081

[B243] Terrenoire C. Houslay M. D. Baillie G. S. Kass R. S. (2009). The cardiac IKs potassium channel macromolecular complex includes the phosphodiesterase PDE4D3. J. Biol. Chem. 284, 9140–9146. 10.1074/jbc.M805366200 19218243 PMC2666564

[B244] Toischer K. Hartmann N. Wagner S. Fischer T. H. Herting J. Danner B. C. (2013). Role of late sodium current as a potential arrhythmogenic mechanism in the progression of pressure-induced heart disease. J. Mol. Cell Cardiol. 61, 111–122. 10.1016/j.yjmcc.2013.03.021 23570977 PMC3720777

[B245] Trinkle-Mulcahy L. Andersen J. Lam Y. W. Moorhead G. Mann M. Lamond A. I. (2006). Repo-Man recruits PP1 γ to chromatin and is essential for cell viability. J. Cell Biol. 172, 679–692. 10.1083/jcb.200508154 16492807 PMC2063701

[B246] Tsaytler P. Harding H. P. Ron D. Bertolotti A. (2011). Selective inhibition of a regulatory subunit of protein phosphatase 1 restores proteostasis. Science 332, 91–94. 10.1126/science.1201396 21385720

[B247] Ubersax J. A. Ferrell J. E. Jr. (2007). Mechanisms of specificity in protein phosphorylation. Nat. Rev. Mol. Cell Biol. 8, 530–541. 10.1038/nrm2203 17585314

[B248] Ucar A. Gupta S. K. Fiedler J. Erikci E. Kardasinski M. Batkai S. (2012). The miRNA-212/132 family regulates both cardiac hypertrophy and cardiomyocyte autophagy. Nat. Commun. 3, 1078. 10.1038/ncomms2090 23011132 PMC3657998

[B249] Uehata M. Ishizaki T. Satoh H. Ono T. Kawahara T. Morishita T. (1997). Calcium sensitization of smooth muscle mediated by a Rho-associated protein kinase in hypertension. Nature 389, 990–994. 10.1038/40187 9353125

[B250] Vermeulen J. T. Mcguire M. A. Opthof T. Coronel R. De Bakker J. M. Klopping C. (1994). Triggered activity and automaticity in ventricular trabeculae of failing human and rabbit hearts. Cardiovasc. Res. 28, 1547–1554. 10.1093/cvr/28.10.1547 8001044

[B251] von Holtey M. Csermely P. Niggemann J. Eckel J. (1996). Insulin-induced phosphorylation of a 38 kDa DNA-binding protein in ventricular cardiomyocytes: possible implication of nuclear protein phosphatase activity. Mol. Cell. Endocrinol. 120, 107–114. 10.1016/0303-7207(96)03828-2 8832569

[B252] Wagner S. Dybkova N. Rasenack E. C. Jacobshagen C. Fabritz L. Kirchhof P. (2006). Ca^2+^/calmodulin-dependent protein kinase II regulates cardiac Na^+^ channels. J. Clin. Invest. 116, 3127–3138. 10.1172/JCI26620 17124532 PMC1654201

[B253] Walker K. S. Watt P. W. Cohen P. (2000). Phosphorylation of the skeletal muscle glycogen-targetting subunit of protein phosphatase 1 in response to adrenaline in vivo. FEBS Lett. 466, 121–124. 10.1016/S0014-5793(99)01771-8 10648825

[B254] Wang R. H. Liu C. W. Avramis V. I. Berndt N. (2001). Protein phosphatase 1-mediated stimulation of apoptosis is associated with dephosphorylation of the retinoblastoma protein. Oncogene 20, 6111–6122. 10.1038/sj.onc.1204829 11593419

[B255] Wang Y. Tandan S. Hill J. A. (2014). Calcineurin-dependent ion channel regulation in heart. Trends. Cardiovasc. Med. 24, 14–22. 10.1016/j.tcm.2013.05.004 23809405 PMC3830706

[B256] Wanichawan P. Louch W. E. Hortemo K. H. Austbo B. Lunde P. K. Scott J. D. (2011). Full-length cardiac Na^+^/Ca^2+^ exchanger 1 protein is not phosphorylated by protein kinase A. Am. J. Physiol. Cell Physiol. 300, C989–C997. 10.1152/ajpcell.00196.2010 21289289 PMC3093950

[B257] Wei S. K. Ruknudin A. Hanlon S. U. Mccurley J. M. Schulze D. H. Haigney M. C. (2003). Protein kinase A hyperphosphorylation increases basal current but decreases β-adrenergic responsiveness of the sarcolemmal Na^+^-Ca^2+^ exchanger in failing pig myocytes. Circ. Res. 92, 897–903. 10.1161/01.RES.0000069701.19660.14 12676818

[B258] Wei S. K. Ruknudin A. M. Shou M. Mccurley J. M. Hanlon S. U. Elgin E. (2007). Muscarinic modulation of the sodium-calcium exchanger in heart failure. Circulation 115, 1225–1233. 10.1161/circulationaha.106.650416 17339552

[B259] Weiss J. N. Chen P. S. Qu Z. Karagueuzian H. S. Garfinkel A. (2000). Ventricular fibrillation: how do we stop the waves from breaking? Circ. Res. 87, 1103–1107. 10.1161/01.res.87.12.1103 11110766

[B260] Westphal R. S. Tavalin S. J. Lin J. W. Alto N. M. Fraser I. D. Langeberg L. K. (1999). Regulation of NMDA receptors by an associated phosphatase-kinase signaling complex. Science 285, 93–96. 10.1126/science.285.5424.93 10390370

[B261] Wilkins B. J. Dai Y. S. Bueno O. F. Parsons S. A. Xu J. Plank D. M. (2004). Calcineurin/NFAT coupling participates in pathological, but not physiological, cardiac hypertrophy. Circ. Res. 94, 110–118. 10.1161/01.RES.0000109415.17511.18 14656927

[B262] Wittkopper K. Dobrev D. Eschenhagen T. El-Armouche A. (2011). Phosphatase-1 inhibitor-1 in physiological and pathological β-adrenoceptor signalling. Cardiovasc. Res. 91, 392–401. 10.1093/cvr/cvr058 21354993

[B263] Wittkopper K. Eschenhagen T. El-Armouche A. (2010a). Phosphatase-1-inhibitor-1: amplifier or attenuator of catecholaminergic stress? Basic Res. Cardiol. 105, 569–571. 10.1007/s00395-010-0107-2 20526608 PMC2916120

[B264] Wittkopper K. Fabritz L. Neef S. Ort K. R. Grefe C. Unsold B. (2010b). Constitutively active phosphatase inhibitor-1 improves cardiac contractility in young mice but is deleterious after catecholaminergic stress and with aging. J. Clin. Invest. 120, 617–626. 10.1172/jci40545 20071777 PMC2810086

[B265] Wolska B. M. (2009). Calcineurin and cardiac function: is more or less better for the heart? Am. J. Physiol. Heart Circ. Physiol. 297, H1576–H1577. 10.1152/ajpheart.00833.2009 19749162

[B266] Woodgett J. R. Cohen P. (1984). Multisite phosphorylation of glycogen synthase. Molecular basis for the substrate specificity of glycogen synthase kinase-3 and casein kinase-II (glycogen synthase kinase-5). Biochim. Biophys. Acta 788, 339–347. 10.1016/0167-4838(84)90047-5 6087911

[B267] Xiao B. Jiang M. T. Zhao M. Yang D. Sutherland C. Lai F. A. (2005). Characterization of a novel PKA phosphorylation site, serine-2030, reveals no PKA hyperphosphorylation of the cardiac ryanodine receptor in canine heart failure. Circ. Res. 96, 847–855. 10.1161/01.RES.0000163276.26083.e8 15790957

[B268] Xiao B. Zhong G. Obayashi M. Yang D. Chen K. Walsh M. P. (2006). Ser-2030, but not Ser-2808, is the major phosphorylation site in cardiac ryanodine receptors responding to protein kinase A activation upon β-adrenergic stimulation in normal and failing hearts. Biochem. J. 396, 7–16. 10.1042/BJ20060116 16483256 PMC1449991

[B269] Xu H. Ginsburg K. S. Hall D. D. Zimmermann M. Stein I. S. Zhang M. (2010). Targeting of protein phosphatases PP2A and PP2B to the C-terminus of the L-type calcium channel Ca v1.2. Biochemistry 49, 10298–10307. 10.1021/bi101018c 21053940 PMC3075818

[B270] Yamakita Y. Totsukawa G. Yamashiro S. Fry D. Zhang X. Hanks S. K. (1999). Dissociation of FAK/p130(CAS)/c-Src complex during mitosis: role of mitosis-specific serine phosphorylation of FAK. J. Cell Biol. 144, 315–324. 10.1083/jcb.144.2.315 9922457 PMC2132894

[B271] Yang L. Liu G. Zakharov S. I. Bellinger A. M. Mongillo M. Marx S. O. (2007). Protein kinase G phosphorylates Cav1.2 1c and β2 subunits. Circ. Res. 101, 465–474. 10.1161/CIRCRESAHA.107.156976 17626895

[B272] Yang L. Liu G. Zakharov S. I. Morrow J. P. Rybin V. O. Steinberg S. F. (2005). Ser1928 is a common site for Cav1.2 phosphorylation by protein kinase C isoforms. J. Biol. Chem. 280, 207–214. 10.1074/jbc.M410509200 15509562

[B273] Yang T. T. Xiong Q. Enslen H. Davis R. J. Chow C. W. (2002). Phosphorylation of NFATc4 by p38 mitogen-activated protein kinases. Mol. Cell. Biol. 22, 3892–3904. 10.1128/MCB.22.11.3892-3904.2002 11997522 PMC133816

[B274] Yger M. Girault J. A. (2011). DARPP-32, Jack of All Trades... Master of Which? Front. Behav. Neurosci. 5:56. 10.3389/fnbeh.2011.00056 21927600 PMC3168893

[B275] Yin X. Cuello F. Mayr U. Hao Z. Hornshaw M. Ehler E. (2010). Proteomics analysis of the cardiac myofilament subproteome reveals dynamic alterations in phosphatase subunit distribution. Mol. Cell. Proteomics 9, 497–509. 10.1074/mcp.M900275-MCP200 20037178 PMC2849712

[B276] Zhang C. L. Mckinsey T. A. Chang S. Antos C. L. Hill J. A. Olson E. N. (2002). Class II histone deacetylases act as signal-responsive repressors of cardiac hypertrophy. Cell 110, 479–488. 10.1016/S0092-8674(02)00861-9 12202037 PMC4459650

[B277] Zhang H. Makarewich C. A. Kubo H. Wang W. Duran J. M. Li Y. (2012). Hyperphosphorylation of the cardiac ryanodine receptor at serine 2808 is not involved in cardiac dysfunction after myocardial infarction. Circ. Res. 110, 831–840. 10.1161/CIRCRESAHA.111.255158 22302785 PMC3322671

[B278] Zhang Y. H. Hancox J. C. (2009). Regulation of cardiac Na^+^-Ca^2+^ exchanger activity by protein kinase phosphorylation–still a paradox? Cell Calcium 45, 1–10. 10.1016/j.ceca.2008.05.005 18614228

[B279] Zhou J. Yi J. Hu N. George A. L. Jr. Murray K. T. (2000). Activation of protein kinase A modulates trafficking of the human cardiac sodium channel in *Xenopus* oocytes. Circ. Res. 87, 33–38. 10.1161/01.RES.87.1.33 10884369

[B280] Zhu G. Liu Y. Shaw S. (2005). Protein kinase specificity. A strategic collaboration between kinase peptide specificity and substrate recruitment. Cell Cycle 4, 52–56. 10.4161/cc.4.1.1353 15655379

[B281] Zhu J. Shibasaki F. Price R. Guillemot J. C. Yano T. Dotsch V. (1998). Intramolecular masking of nuclear import signal on NF-AT4 by casein kinase I and MEKK1. Cell 93, 851–861. 10.1016/S0092-8674(00)81445-2 9630228

[B282] Zou Y. Liang Y. Gong H. Zhou N. Ma H. Guan A. (2011). Ryanodine receptor type 2 is required for the development of pressure overload-induced cardiac hypertrophy. Hypertension 58, 1099–1110. 10.1161/HYPERTENSIONAHA.111.173500 21986507

[B283] Zylinska L. Guerini D. Gromadzinska E. Lachowicz L. (1998). Protein kinases A and C phosphorylate purified Ca^2+^-ATPase from rat cortex, cerebellum and hippocampus. Biochim. Biophys. Acta 1448, 99–108. 10.1016/S0167-4889(98)00128-1 9824678

[B284] Zylinska L. Soszynski M. (2000). Plasma membrane Ca^2+^-ATPase in excitable and nonexcitable cells. Acta Biochim. Pol. 47, 529–539. 11310957

